# Differential regulatory network-based quantification and prioritization of key genes underlying cancer drug resistance based on time-course RNA-seq data

**DOI:** 10.1371/journal.pcbi.1007435

**Published:** 2019-11-04

**Authors:** Jiajun Zhang, Wenbo Zhu, Qianliang Wang, Jiayu Gu, L. Frank Huang, Xiaoqiang Sun

**Affiliations:** 1 School of Mathematics, Sun Yat-Sen University, Guangzhou, China; 2 Department of Pharmacology, Zhongshan School of Medicine, Sun Yat-Sen University, Guangzhou, China; 3 Brain Tumor Center, Division of Experimental Hematology and Cancer Biology, Cincinnati Children’s Hospital Medical Center, Cincinnati, OH, United States of America; 4 Department of Pediatrics, University of Cincinnati College of Medicine, Cincinnati, OH, United States of America; 5 Department of Medical Informatics, Zhongshan School of Medicine, Sun Yat-Sen University, Guangzhou, China; Key Laboratory of Tropical Disease Control (Sun Yat-Sen University), Chinese Ministry of Education, Guangzhou, Guangdong, China; University of California Irvine, UNITED STATES

## Abstract

Drug resistance is a major cause for the failure of cancer chemotherapy or targeted therapy. However, the molecular regulatory mechanisms controlling the dynamic evolvement of drug resistance remain poorly understood. Thus, it is important to develop methods for identifying key gene regulatory mechanisms of the resistance to specific drugs. In this study, we developed a data-driven computational framework, DryNetMC, using a differential regulatory network-based modeling and characterization strategy to quantify and prioritize key genes underlying cancer drug resistance. The DryNetMC does not only infer gene regulatory networks (GRNs) via an integrated approach, but also characterizes and quantifies dynamical network properties for measuring node importance. We used time-course RNA-seq data from glioma cells treated with dbcAMP (a cAMP activator) as a realistic case to reconstruct the GRNs for sensitive and resistant cells. Based on a novel node importance index that comprehensively quantifies network topology, network entropy and expression dynamics, the top ranked genes were verified to be predictive of the drug sensitivities of different glioma cell lines, in comparison with other existing methods. The proposed method provides a quantitative approach to gain insights into the dynamic adaptation and regulatory mechanisms of cancer drug resistance and sheds light on the design of novel biomarkers or targets for predicting or overcoming drug resistance.

## Author summary

Leveraging global gene expression patterns to study dynamical mechanisms of cancer drug resistance is an appealing yet challenging task for both experimental and computational biologists. In this study, we proposed a dynamic network-based computational method to prioritize the key genes responsible for cancer drug resistance, which is significantly innovative compared to the conventional differential expression method and co-expression network-based differential analysis. In addition, our method is verified to be more accurate compared with several state-of-the-art methods used for inferring GRNs. We applied our computational method to a set of time-course RNA-seq data of gliomas, and several novel predictions were verified by the additional gene expression/cellular response data or the clinical data. Our study provides a principled approach to gain insights into dynamic adaptation and regulatory network mechanisms of cancer drug resistance, and sheds lights on designing new biomarkers or targets for predicting or controlling drug resistance.

## Introduction

Drug resistance is often an inevitable event that limits the effectiveness of cancer chemotherapy or targeted therapy. Unraveling key genes and their regulatory mechanisms controlling the acquisition and development of drug resistance remains a challenging task. Therefore, it is important to develop systematic approaches for identifying key gene regulatory mechanisms underlying the resistance of cancer cells to specific drugs or therapeutics.

Our preliminary studies have established an experimental model for the glioma differentiation therapy with cAMP activators [1], which enables us to test the differential sensitivities of various glioma cell lines to the differentiation agents. In the presence of a cAMP activator (e.g., dbcAMP), glioma cells can differentiate into astroglia cells [2]. Importantly, different glioma cell lines show differential sensitivities to treatment with a cAMP activator [1]. An analysis of the morphological changes experienced by glioma cells demonstrated that some cell lines, but not others, differentiated, which indicates the existence and impact of drug resistance. However, the key regulators of resistance to drugs in glioma differentiation therapy remain unknown.

As a result, distinguishing cellular states between drug sensitivity and drug resistance at the molecular level is critical for the study of drug resistance. The conventional method involves the use of a set of differentially expressed genes to distinguish between drug sensitivity and resistance [3, 4], but this approach ignores the interactions between genes.

In recent years, network-based approaches [5–8] have been developed to identify important gene sets underlying drug resistance. However, most of the developed correlation network-based or undirected graph-based methods [9–14] and their applications [15, 16] do not consider the dynamic changes in gene expression and thus often ignore the regulatory directions or causal relationships between genes. In fact, the acquisition and development of cancer drug resistance are always dynamic processes characterized by temporal changes in gene expression. Therefore, a global expression data-based computational approach that utilizes gene interaction information and expression dynamics could be developed to model and characterize the gene regulatory networks (GRNs) underlying cancer drug resistance.

In this study, we developed a time-course RNA-seq data-driven dynamical network modeling and characterization method to quantify and prioritize key genes for predicting drug sensitivities and designing potential targets against drug resistance. The advances and novelty of our method include not only accurate reconstruction of GRNs via an integrated approach but also novel characterization and quantification of GRN properties for measuring and ranking node importance.

We used glioma differentiation therapy as a realistic case and time-course RNA-seq to investigate the temporal gene expression changes in sensitive and resistant cells. We then reconstructed GRNs for both sensitive cells and resistant cells and analyzed their difference in network topology, local network entropy and expression dynamics. We further designed a novel quantification method to prioritize the most important genes in the differential network that are responsible for drug resistance by considering the network topology, network entropy and adaptation dynamics. The clinical data verified that the top-ranked genes were associated with the targeted therapeutic response and prognosis of glioma patients. Furthermore, the *in vitro* experimental data were employed to validate the drug sensitivity prediction based on the similarity of the temporal patterns of the prioritized key genes. We compared our method with other methods including the conventional differential expression analysis and differential co-expression network–based method. The computational method developed in this study is generally applicable for the analysis of time-course RNA-seq data designed for studying drug resistance in many cancer types.

## Materials and methods

**Fig 1:**
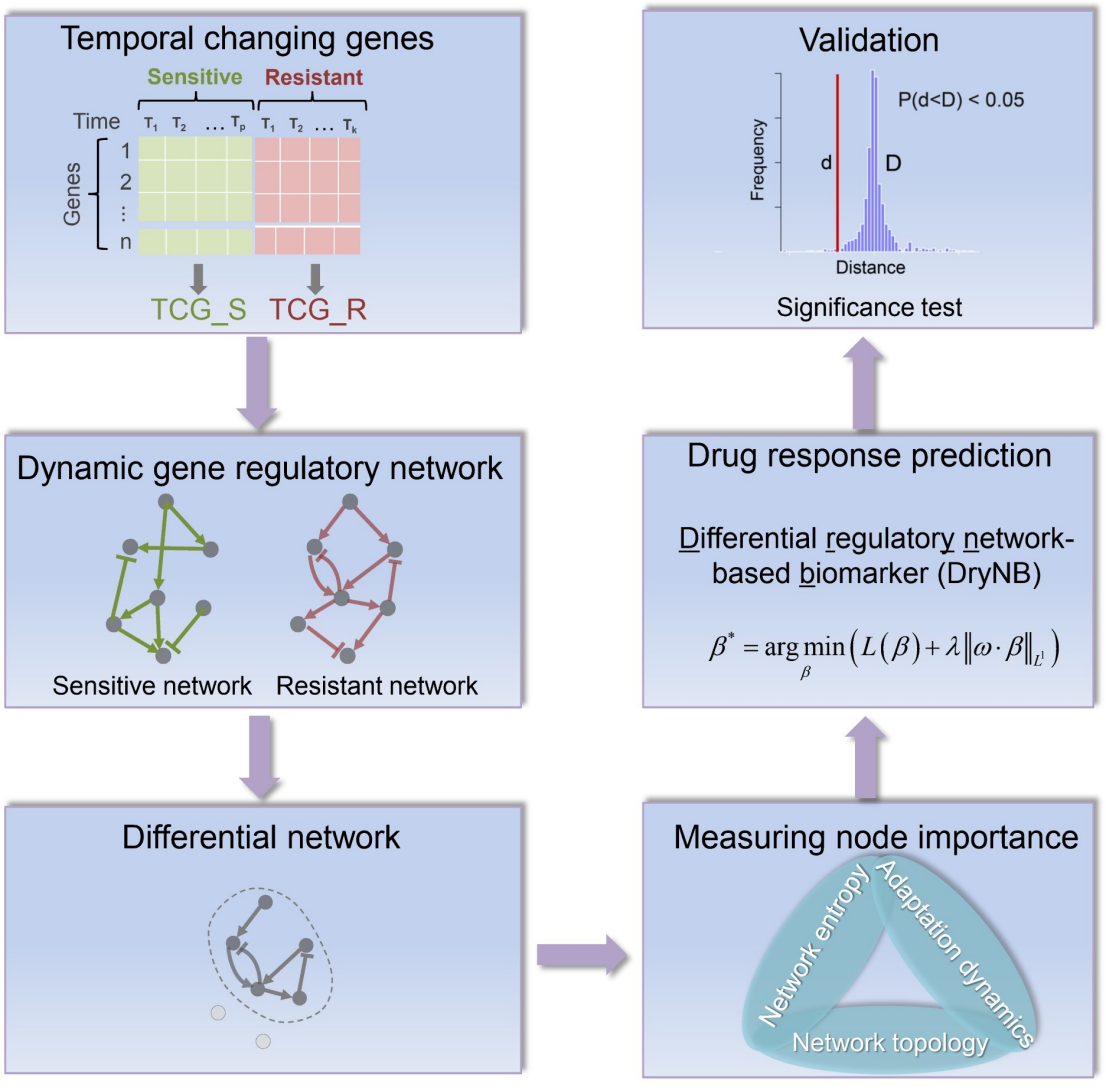
The computational method of DryNetMC (differential regulatory network-based modeling and characterization) developed to prioritize key genes responsible for drug resistance. (I) The TCGs were selected as core genes from time-course RNA-seq data of sensitive and resistant cells. (II) The dynamic GRNs for sensitive cells and resistant cells were reconstructed via an integrated approach that incorporates prior information, data interpolation, dynamic systems modeling and regularized regression methods. (III) Subsequently, a differential network was then extracted and its functional enrichment was performed. (IV) Moreover, the features of network topology, local entropy and adaptation dynamics were analyzed to measure the importance of each node in the differential network for prioritizing key genes responsible for drug resistance. (V) In addition, the above node importance measurement was incorporated into a differential regulatory network-based biomarker (DryNB) model for predicting drug response of clinical patients. (VI) Furthermore, experimental data and statistical significance test were used to validate the effectiveness of the key genes prioritized by DryNetMC.

The computational pipeline for the time-course transcriptome-based modeling and characterization of the GRNs underlying drug resistance is illustrated in **Fig 1**. Below, we describe the details of each step.

### Identification of temporal changes in gene expression

The RNA-seq data for both sensitive cells and resistant cells were measured at *T*_1_, *T*_2_, …, and *T*_*K*_ following drug treatment. The raw RNA-seq reads were processed using a standard pipeline [17–20] (see details in **S1 Text**). Because gene expressions show temporal changes over time, we designed the following algorithm to globally select significant temporally changing genes (TCGs) by comparing expression levels between any two time points for a given gene. A given gene with expression level *u*_*k*_ (*k* = 0, 1, …, *K*) was defined as a significant TCG if the following criteria were satisfied:

- $\underset{k}{\max}(u_{k})\geq \zeta$;
- $\frac{u_{j}}{u_{k}}\geq \delta$ or $\frac{u_{j}}{u_{k}}\leq \frac{1}{\delta }$ for some *j* and *k*.

In other words, a given gene was defined as a TCG if the maximal expression level of the gene was greater than *ζ* and the fold change of its expression level between two time points was greater than *δ*. *ζ* and *δ* could be chosen according to the percentage or the number of the selected TCGs for further network construction. We empirically select the first hundreds of TCGs (~5%-10% genes of the whole transcriptome) for the following network modeling and visualization. As such, thresholds for *ζ* and *δ* can be set accordingly. In this study, *ζ* was set to 10 and *δ* was set to 5, as in our previous study [21].

### Data interpolation

Given that the total number of time points (i.e., *K*+1) of RNA-seq data in practice is often relatively small, we sampled more data points of the TCG expression data using a Hermit polynomial interpolation method [22]. The piecewise cubic Hermit interpolation polynomial *x*(*t*) was constructed to approximate the gene expression level *u*_*k*_ (*k* = 0, 1, …, *K*) on each subinterval [*T*_*i*_, *T*_*i*+1_] (*i =* 0, 1, …, *K*-1), such that *x*(*T*_*k*_) = *u*_*k*_ and the derivative of *x*(*t*) is continuous. The details of construction of piecewise cubic Hermit interpolation polynomial is provided in **S1 Text**. We chose this method because *x*(*t*) preserves the monotonicity, local extremum and nonnegativity of the expression data. We noted that other interpolation methods, such as polynomial spline or cubic spline, might result in unrealistic negative interpolated values and unexpected variations in gene expression. We then uniformly took *n* (for example, *n* = 100) points from *x*(*t*), denoting them as *x*(*t*_1_), *x*(*t*_2_), …*x*(*t*_*n*_).

### Modeling and reconstruction of the GRNs

We next inferred the causal relationship between each pair of nodes using time-series gene expression data from sensitive cells and resistant cells. We used a set of ordinary differential equations (ODEs) to model the dynamic gene-regulatory network as follows:

$$\frac{dx_{i}}{dt}=f_{i}\left(x_{1},x_{2},\cdots ,x_{L}\right),\;i=1,2,\cdots ,L$$ (1)

where *x*_*i*_(*t*) represents the continuous expression of gene *i* at time *t*, *L* is the number of nodes in the network. The function *f*_i_ can be linear, piece-wise linear or nonlinear. To reduce the model complexity, we assumed that

$$f_{i}(x_{1},x_{2},\cdots ,x_{L})=\sum_{j=1}^{L}e_{ij}a_{ij}x_{j}+b_{i},\;i=1,2,\cdots ,L$$ (2)

where *a*_*ij*_ is the interaction strength from *x*_*j*_ to *x*_*i*_, and *b*_*i*_ is a constant number accounting for the effects of degradation or self-activation. *e*_*ij*_ represents the prior information and association between gene *i* and gene *j* in an initial correlation network *G*(*V*, *E*) (see details in **S1 Text**).

We approximate $\frac{dx_{i}}{dt}\left(t_{k}\right)\approx \frac{x_{i}(t_{k+1})-x_{i}(t_{k})}{t_{k+1}-t_{k}}$ and denote $y_{i}(t_{k})=\frac{x_{i}(t_{k+1})-x_{i}(t_{k})}{t_{k+1}-t_{k}}$, where *t*_*k*+1_−*t*_*k*_ is sufficiently small (since *n* is chosen large enough as mentioned above). Therefore, the above continuous model (i.e., Eq (2)) can be rewritten as

$$y_{i}\approx \sum_{j=1}^{L}e_{ij}a_{ij}x_{j}+b_{i},\;i=1,2,\cdots ,L$$ (3)

We then denote *Y* = (*Y*_*i*,*k*_)_*L*×*K*_ = (*y*_*i*_(*t*_*k*_))_*L*×*K*_, *X* = (*X*_*j*,*k*_)_*L*×*K*_ = (*x*_*j*_(*t*_*k*_))_*L*×*K*_, *E* = (*e*_*ij*_)_*L*×*L*_, *A* = (*a*_*ij*_)_*L*×*L*_, and *B* = *diag*(*b*_*i*_). Eq (3) can be transformed into the following linear regression problem:

Y=(E∘A)X+B+ε (4)

where *ε* = (*ε*_1_,*ε*_2_,⋯,*ε*_*L*_)^*T*^ is the error term. *ε*_1_, *ε*_2_, ⋯, *ε*_*L*_ are mutually independent normal random variables with means 0, 0, ⋯, 0 and variances *σ*_1_, *σ*_2_, ⋯, *σ*_*L*_, respectively. Note that *E*∘*A* represents the Hadamard product of *E* and *A*.

We then estimated the model parameters, i.e., gene interaction matrix *A* and *B*, by fitting the model to the experimental data (*X*^*exp*^ and *Y*^*exp*^) using a regularized regression method (e.g., LASSO [23]) as follows:

$$\underset{A,B}{\min}\left({\left\|Y_{i,\cdot }{}^{exp}-{\left(E\circ A\right)}_{i,\cdot }X^{\exp}-B_{i,\cdot }\right\|}_{L^{2}}^{2}+\lambda_{i}{\left\|A_{i,\cdot }\right\|}_{L^{1}}^{}\right),\;i=1,2,\cdots ,L$$ (5)

where *λ*_*i*_ is the penalty weight. Ten-fold cross validation was performed to select the optimal value of *λ*_*i*_ that minimizes the mean cross-validated errors. The regression coefficients at the optimal penalty weight were computed as parameter estimations. The above regularized regression model was solved using the R package "glmnet" [24].

To determine whether an edge in the GRN model is redundant, we examined the impact of removing an edge on the model fitting result. We defined a strength threshold, *θ*, to select the significant edges in the network, i.e., the edge from gene *j* to *i* was preserved if |*a*_*ij*_|≥*θ* and was deleted if |*a*_*ij*_|<*θ*. The Bayesian information criterion (BIC) was employed to quantify the trade-off between the goodness-of-fit and the complexity of the network model. For a network with *p* nonzero edges fitted to the experimental data with *N* (= *L*×*K*) samples, the BIC was calculated as follows [25]:

$$BIC=N\cdot \log\left(\frac{1}{N}{\left\|Y^{\exp}-\left(E\circ A\right)X^{\exp}-B\right\|}_{L^{2}}^{2}\right)+p\cdot \log\left(N\right)$$ (6)

Given a series of *θ* and thus different edge numbers, the lowest BIC identifies preferred model that possesses both good predictive power and network simplicity.

### Differential network analysis

*G*(V*, *E*^*S*^) and *G*(V*, *E*^*R*^) denote the sensitive and resistant networks derived from the above method, respectively. We then defined a differential network between the resistant (*E*^*R*^) and sensitive (*E*^*S*^) networks as *D* = *G*(V*, *E*^*R*^*/E*^*S*^), and $({\hat{a}}_{ij}{)}_{m\times n}$ denotes the regulatory coefficient matrix of the differential network. The networks were visualized using the DyNet app in Cytoscape [26].

The genes in the differential network were inputted into the Gene Ontology (GO) knowledgebase (http://www.geneontology.org/) [27] for functional pathway enrichment analysis, and the significantly enriched biological processes were extracted. Gene annotation was performed using the GeneCards database (http://www.genecards.org/) [28].

### Measurement of node importance

We subsequently sought to measure the importance of each node in the differential network by considering the network topology, interaction strengths and gene expression dynamics. We defined the hub score, local network entropy and adaptation score for each node as follows and then integrated these values into a single comprehensive index.

#### (1) *Hub score*

We first considered the network topology and defined a hub score for each node in the differential network *D* to estimate the value of its links to other nodes. $\tilde{A}$ is the adjacent matrix of *D*, and we subjected $\tilde{A}$ to singular value decomposition. We calculated the principal eigenvector of *AA*^*T*^ and denoted it *H =* (*h*_*1*_, *h*_*2*_, …, *h*_*L*_). The hub score of node *i* was defined as *h*_*i*_.

#### (2) *Local network entropy*

By viewing the GRN as a random walk of information flow [29], we defined local network entropy to quantify the degree of randomness in the local pattern of information flux around each node. Based on the interaction matrix *A* = (*a*_*ij*_)_*L×L*_, the entropy of node *i* in the GRN was defined as follows:

$$S_{i}=-\sum_{j\in N(i)}p_{ij}\;\log_{2}(p_{ij})$$ (7)

where $p_{ij}=\left|a_{ij}\right|/\sum_{j=1}^{N(i)}\left|a_{ij}\right|$ and *N*(*i*) is the set of all neighborhood of node *i*. Higher entropy indicates that the gene network is more robust with respect to the perturbations. The entropy change score for the genes in the differential network was defined as

$$\Delta S_{i}=S_{i}^{R}-S_{i}^{S}$$ (8)

where $S_{i}^{S}$ and $S_{i}^{R}$ denote the entropy of gene *i* in the sensitive and resistant networks, respectively.

#### (3) *Adaptation score*

We calculated the relative response of each gene by measuring the dynamic change in gene expression as follows:

$$R_{i}=\frac{x_{i}(T)-x_{i}(0)}{x_{i}(0)}$$ (9)

where *T* was 48 hr in our experiment. To quantify the adaptation dynamics of each gene in the differential network, we defined the following adaptation score:

$$D_{i}=\frac{x_{i}^{R}(T)-x_{i}^{R}(0)}{x_{i}^{R}(0)}/\frac{x_{i}^{S}(T)-x_{i}^{S}(0)}{x_{i}^{S}(0)}$$ (10)

where $x_{i}^{S}(t)$ and $x_{i}^{R}(t)$ represent the dynamic expression levels of gene *i* in the sensitive and resistant networks, respectively. If gene *i* presented a more adaptive response to the drug treatment in the resistant cells than in the sensitive cells, a high adaptation score was obtained.

We ranked the hub score (*H*), entropy (*S*) and adaptation score (*D*) and denoted their rank values (dimensionless) for each node as $r_{i}^{H},\;r_{i}^{S}$ and $r_{i}^{D}$, respectively. We then defined an importance score (*I*_*i*_) for each node as the normalized rank sum of these values:

$$I_{i}=\frac{r_{i}^{H}+r_{i}^{S}+r_{i}^{D}}{\sum_{i=1}^{L}\left(r_{i}^{H}+r_{i}^{S}+r_{i}^{D}\right)}$$ (11)

The rankings of the importance score were used to prioritize key genes responsible for the drug resistance.

### Assessment of clinical relevance of the top ranked genes

We hypothesized that the top ranked genes according to the above-described node importance score were clinically relevant to the response of glioma patients to targeted therapeutics. Therefore, we collected related clinical data and defined a differential regulatory network-based biomarker (DryNB) to select the genes with the most clinical relevance (see **S1 Text**). The DryNB incorporated the node importance measurement (Eq (11)) as a penalty weight in a logistic regression model. A drug-sensitivity score was formulated to predict the response of glioma patients to the targeted therapies based on the expression levels of selected genes. The TCGA samples of glioma patients who received targeted therapy (N = 289) were randomly divided into training and test subdatasets, and the areas under the ROC curves (AUCs) were computed. In addition, the association of the prioritized genes with the survival of glioma patients (N = 610) was assessed through K-M analysis based on the Cox model [30].

### Experimental validation

The temporal expression profiles of the prioritized genes were validated by performing qPCR experiments (**S2 Text**), and their differential expression between normal and tumor tissues was assessed using the patient data. Additional RNA-seq data from another glioma cell line, U87MG, was obtained in this study to test the responses of these cells to dbcAMP treatment, verified by morphological changes of U87MG cells [1].

## Results

### Distinct temporal gene expression patterns of sensitive and resistant cells

Our experimental data (**S1 Fig**) demonstrated that two glioma cell lines, DBTRG-05MG and LN-18 cells, showed differential sensitivities to treatment with dbcAMP, an activator of cAMP. In the following text, we refer to DBTRG-05MG and LN-18 cells as sensitive and resistant cells, respectively. The RNA-seq data for both sensitive cells and resistant cells were measured at 0, 6, 12, 24 and 48 hr following drug treatment. The first time point (i.e., 0 hr) corresponds to no treatment condition.

**Fig 2:**
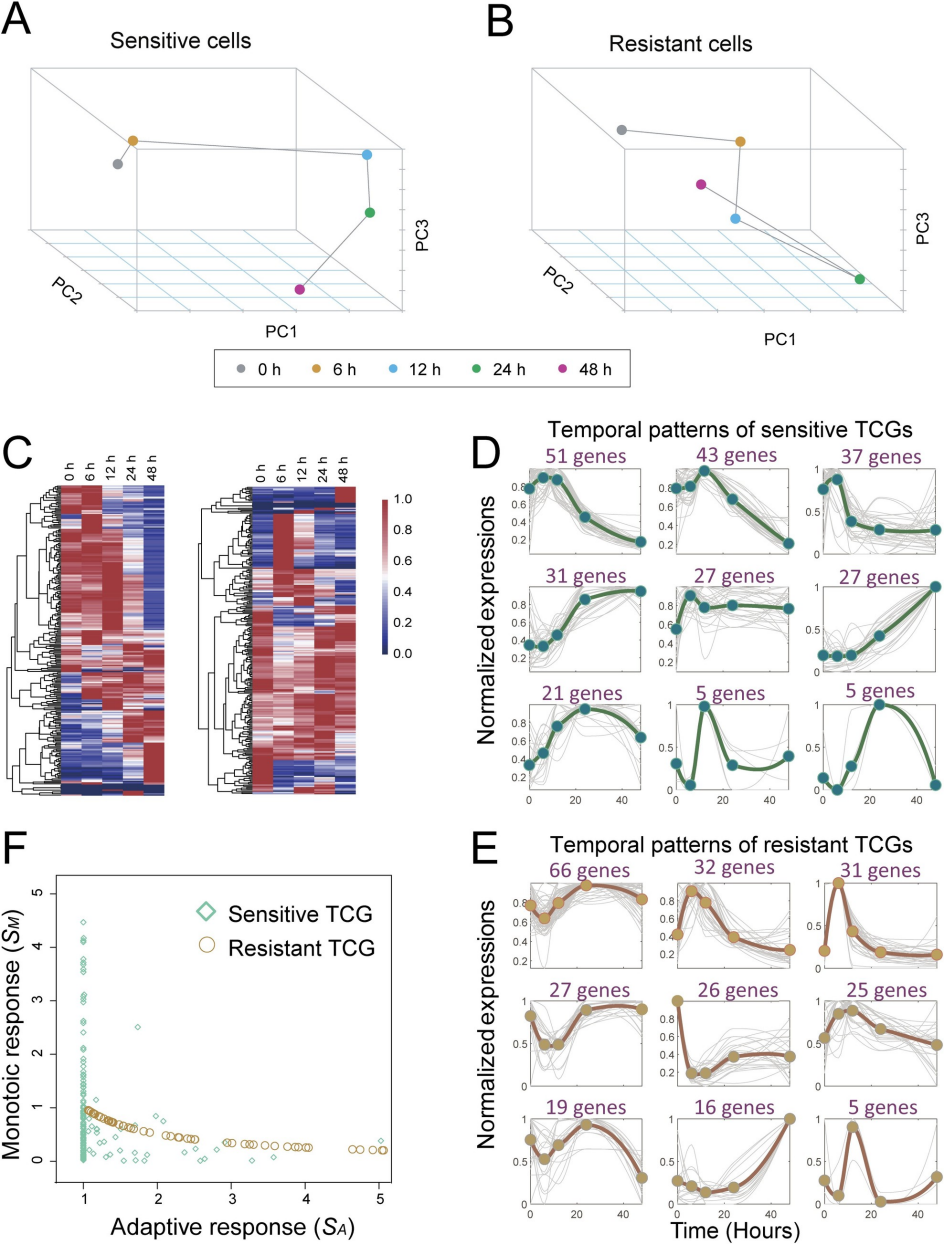
Distinct temporal gene expression profiles and patterns of dbcAMP-sensitive and dbcAMP-resistant glioma cells. (**A-B**) Principal component analysis of RNA-seq transcriptomic data from the sensitive and resistant cells. The resistant cells showed an adaptive response, whereas the sensitive cells did not show this type of response. (**C**) Heat maps showing the distinct expression profiles of temporal changing genes (TCGs) in the sensitive (left panel) and resistant cells (right panel). (**D-E**) Clustering of the dynamic trends of the TCGs in the sensitive and resistant cells. The TCG profiles were divided into nine clusters. Most TCGs in the sensitive cells showed monotonic (increasing or decreasing) patterns (D), whereas most TCGs in the resistant cells exhibited adaptive dynamics (E). (**F**) Scatter plot showing scores for monotonic response (*S*_*A*_) and adaptive response (*S*_*M*_) of TCGs in the sensitive cells and resistant cells, respectively. The TCGs in the resistant cells tended to have a higher adaptive response score but a lower monotonic response score compared to the TCGs in the resistant cells. The assessment of statistical significance was given in S2C and S2D Fig.

A principal component analysis (PCA) of the temporal expression of all the genes in the sensitive and resistant cells (**Fig 2A and 2B**) demonstrated that these showed different phenotypic trajectories. The cellular states of sensitive cells changed over time and went far from their starting point, whereas the resistant cells first moved away and then returned closer to the starting point. This result suggested that the resistant cells show a dynamic adaptive response under dbcAMP treatment.

We selected the significant TCGs to analyze their temporal expression patterns. **Fig 2C** shows the expression profiles of TCGs in the sensitive and resistant cells. The TCG profiles were divided into nine clusters using the k-means clustering method (**Fig 2D and 2E**). Most TCGs in the sensitive cells showed "monotonic response" patterns in which the gene expression exhibited an ascending or descending trend (**Fig 2D**), whereas most TCGs in the resistant cells exhibited "adaptive response" patterns (**Fig 2E**), in which the gene expression temporally increased or decreased at an early stage, followed by a return to near-baseline levels at the late stage. Furthermore, we quantitatively evaluated the tendencies of monotonic response or adaptive response of the TCGs (**S2A and S2B Fig**). Clearly, the TCGs in the resistant cells tended to have a higher adaptive response score but a lower monotonic response score compared to the TCGs in the resistant cells (**Fig 2F**), with statistical significance assessed in **S2C and S2D Fig**.

### Dynamic modeling and reconstruction of sensitive and resistant GRNs

Prior to working with our realistic RNA-seq data, we tested the effectiveness of the DryNetMC with respect to GRN inference using a synthetic dataset (**S3 Fig**). Time-course gene expression data were simulated using a set of ODEs based on a five-node true network with typical network motifs (e.g., feedback loops and crosstalk) (**S3A and S3B Fig**). Sampling data at 0, 6, 12, 24, and 48 hr (mimicking the experimental measurements) were obtained and Hermit interpolations were performed (**S3C Fig**). The performance of the DryNetMC with respect to GRN inference was compared with several existing methods, including PCC-based correlation network method (PCCNet) [31], tree-based ensemble learning methods (GENIE3 [32]), a dynamic Bayesian network method (GRENITS) [33], the ODE-LASSO method (OdeLasso) and the ODE modeling method incorporating prior network information (OdeLassoP) [34, 35]. The area under the curve (AUC) of ROC (**S3D Fig**) of the DryNetMC is notably greater than that of the other methods. Moreover, we compared the performance of the DryNetMC with the other methods on 100 simulated datasets (see details in **S1 Text**). The comparison of the AUC values (**S4 Fig**) demonstrated that the DryNetMC significantly outperformed the other methods.

We then used real RNA-seq data from DBTRG-05MG and LN-18 cells treated with dbcAMP to reconstruct the sensitive and resistant networks using the dynamic modeling method. **S5 Fig** shows the parameter estimation and edge selection results from the network reconstruction. **S5A** and **S5B Fig** show the frequency distributions of the penalty weights in the LASSO regression for the sensitive and resistant networks, respectively. **S5C and S5D Fig** show the frequency distributions of the mean cross-validation errors. Most of the mean cross-validation errors were less than 0.003, indicating good accuracy of the fitting and reliable network reconstruction. **S5E and S5F Fig** demonstrate the change in the BIC values against the absolute values of edge strength (*θ*). The minimal absolute values of edge strength were achieved at approximately 0.01. Therefore, we used 0.01 as the strength threshold to select the significant edges in the networks (see also **S6 Fig**).

**Fig 3:**
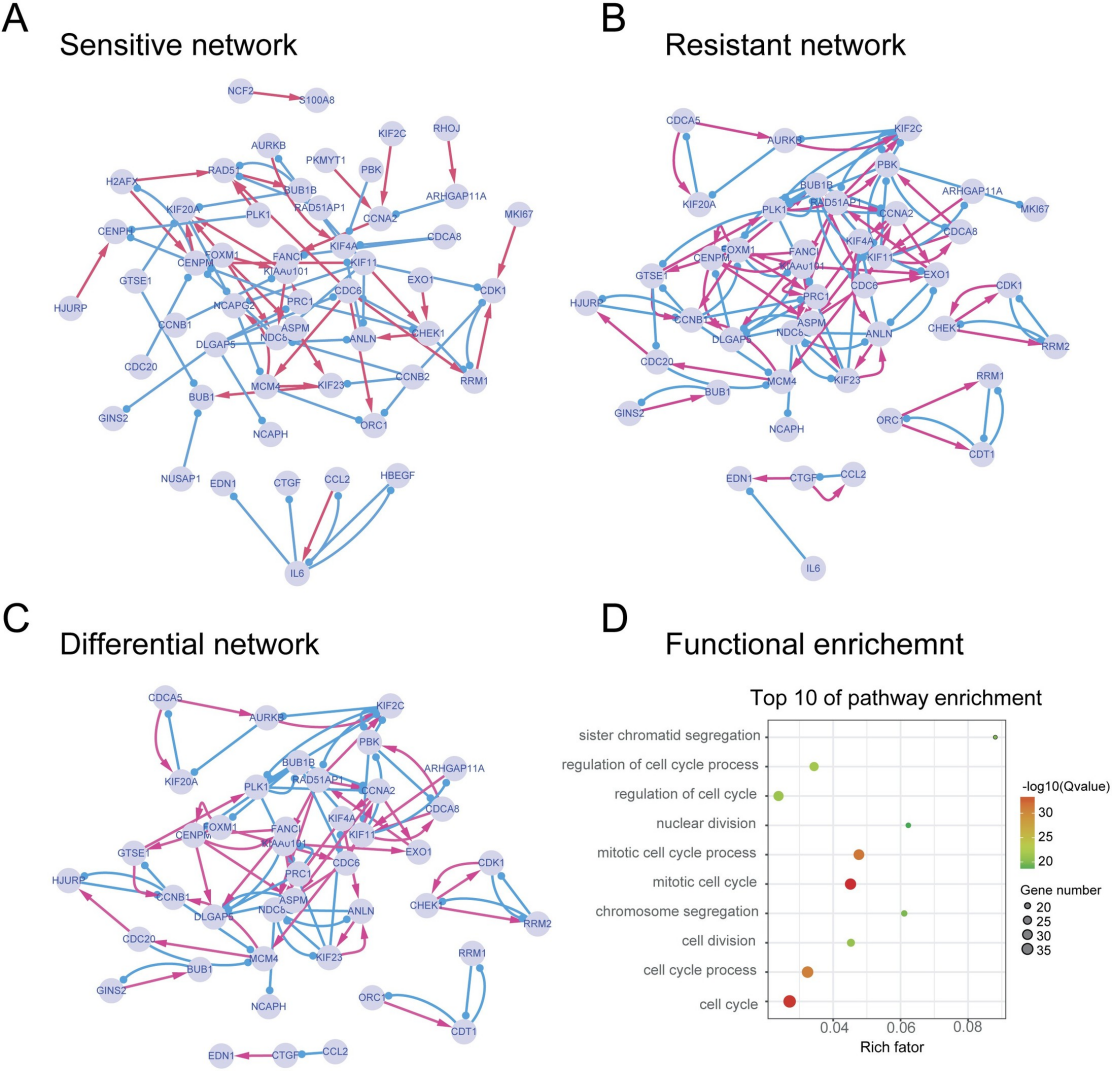
The reconstructed sensitive and resistant networks as well as the differential network. **(A)** Sensitive GRN. **(B)** Resistant GRN. (**C**) The differential network. The genes are represented as circled nodes, and the activating and repressing interactions are represented as red arrows and blue lines, respectively. (**D**) Top 10 significantly enriched pathways involving genes in the differential network. Cell cycle and chromosome segregation were significantly enriched.

**Fig 3A and 3B** visualize the reconstructed GRNs for the sensitive and resistant cells. The genes are represented as circled nodes, and the activating and repressing interactions are represented as red arrows and blue lines, respectively. The sensitive and resistant networks showed different connections between most pairs of genes, suggesting that different gene regulations underlie the sensitive and resistant cellular states.

We defined a differential network (**Fig 3C**) by reserving edges in the resistant network that should be specifically responsible for the resistant cellular state. The functional enrichment of genes involved in the differential network revealed that cell cycle and chromosome segregation were crucial pathways, which suggested that these biological processes were significantly involved in drug resistance during glioma differentiation therapy (**Fig 3D**).

### Characterization of topology, entropy and dynamics of sensitive and resistant GRNs

**Fig 4:**
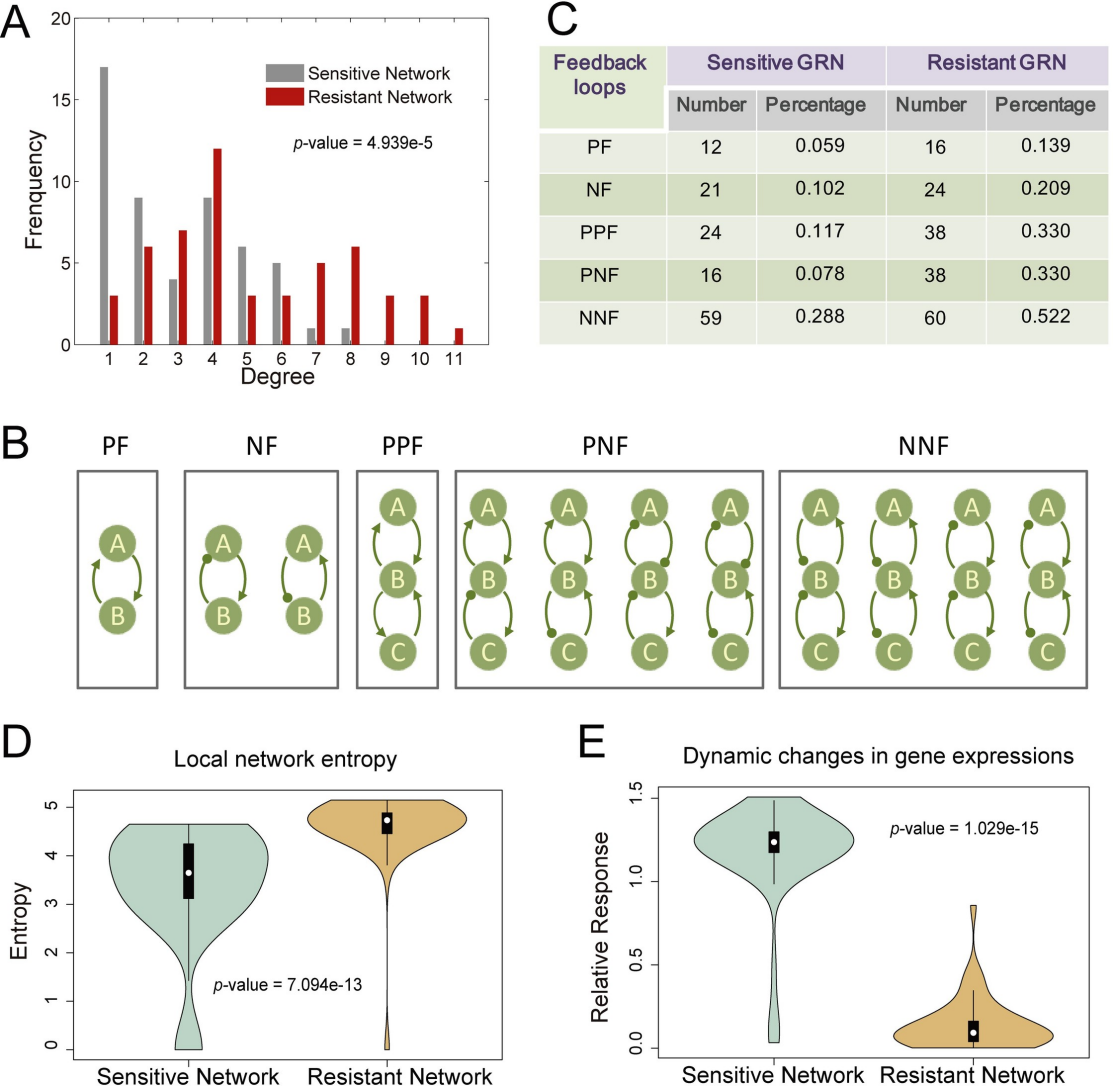
Characterization of the difference between sensitive and resistant GRNs. (**A**) Degree distributions of the sensitive and resistant networks. One-tailed Wilcoxon rank sum test p-value = 4.939×10^−5^. (**B**) Two- and three-node feedback loops involved in the GRNs. PF: positive feedback; NF: negative feedback; PPF: positive-positive feedback; PNF: positive-negative feedback; NNF: negative-negative feedback. (**C**) The percentages of various feedback loops in the resistant network were substantially larger than those in the sensitive network. (**D-E**) Violin plots comparing the differences of local network entropies (D) and dynamic changes in gene expression (E) between the sensitive and resistant networks, with one-tailed Wilcoxon rank sum test p-values of 7.094×10^−13^ and 1.029×10^−15^, respectively.

We examined the topological difference between the sensitive and resistant GRNs. As shown in **Fig 4A**, the node degree of the resistant network (red bars) was significantly larger than that of the sensitive network (gray bars). We also investigated two- and three-node feedback motifs, including positive feedback (PF), negative feedback (NF), positive-positive feedback (PPF), positive-negative feedback (PNF), and negative-negative feedback (NNF) (**Fig 4B**). We then measured the number and percentage of various feedback loops in the sensitive and resistant networks (**Fig 4C**), which indicated that the percentage of various feedback loops in the resistant network was substantially higher than that in the sensitive network. These results revealed that the genes exhibited more complex regulatory relationships in the resistant cells, which enabled the resistant cells to show increased robustness in response to the drug attack.

In addition, by viewing the GRN as a random walk of information flow [29], we defined the local network entropy to quantify the randomness of information flux around each gene based on the interaction strengths of the sensitive and resistant networks. As shown in **Fig 4D,** the local network entropy was increased in the resistant network compared with the sensitive network, which indicated the increased robustness of the resistant network with respect to perturbations. This result is consistent with the dynamic changes in gene expression in the two networks in response to dbcAMP treatment (**Fig 4E)**. The genes in the resistant network exhibited less changes compared with those in the sensitive network, which supported the notion that the resistant network was more robust to external perturbations.

### Measurement of the importance of nodes for prioritizing key genes underlying drug resistance

Based on the above network characterization, we defined and computed the hub score, entropy and adaptation score for each gene in the differential network (see Methods section) to measure the node importance and thereby identify key genes responsible for drug resistance, which might be explored as potential drug targets. **S1 Table** lists the hub score, entropy and adaptation score as well as their ranking numbers for each node. The normalized rank sum (*I*_*i*_) (Eq (11)) was accordingly calculated to define the importance score of each gene. **S2 Table** lists the annotation for each gene ordered by the node importance score.

To test the functional roles of the top-ranked genes in the drug resistance of glioma patients and their clinical relevance, we developed a DryNB model through incorporation of node importance measurement into a logistic model (**S1 Text**). The identified DryNB included seven genes (KIF2C, CCNA2, NDC80, KIF11, KIF23, ANLN and CENPM) that were significantly associated with drug sensitivity or resistance of glioma patients to the targeted therapies.

**Fig 5:**
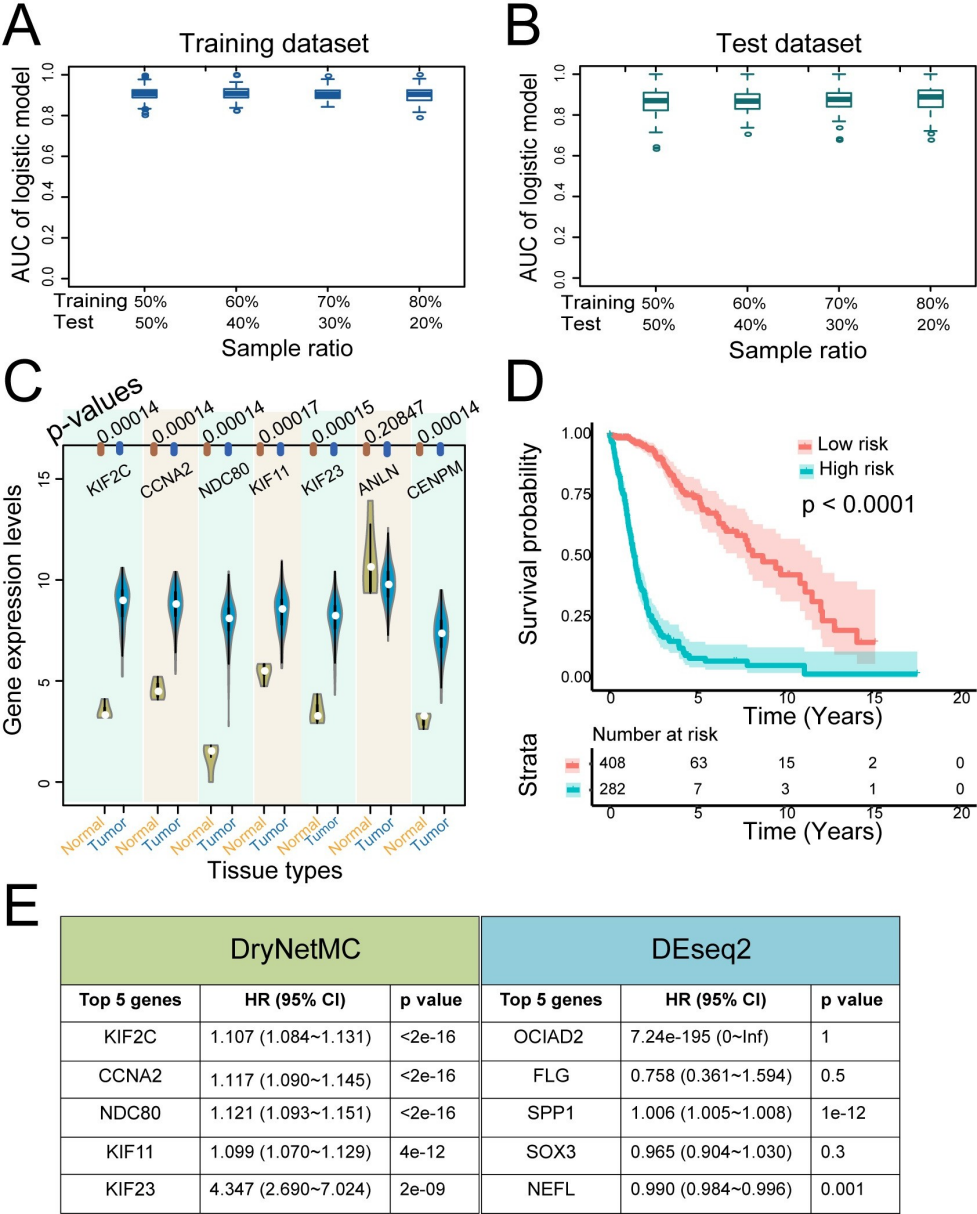
The top-ranked genes in the differential network were predictive of the targeted therapeutic response and prognosis of glioma patients. (**A-B**) The areas under the ROC curves (AUCs) were computed for both the training datasets (A) and the test datasets (B) of glioma patients. (**C**) Differential expression profiles of the seven identified genes in the normal and tumor tissues of glioma patients. The p-values were assessed using the one-tailed Wilcoxon rank sum test. (**D**) The seven identified genes were associated with the survival probability of glioma patients (N = 610). A log rank test was used to assess the significance between two curves, with a p-value less than 0.0001. (**E**) Statistical significance of the association of the five top-ranked genes identified by the DryNetMC with prognosis of glioma patients, in comparison with a conventional differential expression analysis method, DEseq2. Wald test p-value was used to assess the prognostic significance of each gene in the table.

The TCGA samples of glioma patients who received targeted therapies were randomly divided into the training and test subdatasets based on several different sample ratios to the total sample number (N = 289). The sample division at each ratio was repeated 100 times. The areas under the ROC curves (AUCs) were computed for both the training dataset (**Fig 5A**) and the test dataset (**Fig 5B**), which demonstrated good accuracy of the DryNB for predicting drug response. In addition, the seven identified genes showed differential expression profiles in the normal and tumor tissues of glioma patients (**Fig 5C**). **Fig 5D** further shows the significant association of the identified seven genes with the survival probability of glioma patients based on the data from the TCGA samples (N = 610). These results demonstrated the significant clinical relevance of the identified genes and verified their important roles in drug resistance and cancer progression.

Notably, the five top-ranked genes (KIF2C, CCNA2, NDC80, KIF11 and KIF23) were consistently included in the DryNB. We further compared the significance of the clinical relevance of the top-ranked genes identified respectively from the DryNetMC and the conventional differential expression method (e.g., DEseq2 [36]). We used univariate Cox proportional hazards model [30] to assess the association of each gene with prognosis of glioma patients based on an additional CGGA dataset (N = 310). Hazard ratio (HR) and Wald test p-value were computed for each gene. All five top-ranked genes (KIF2C, CCNA2, NDC80, KIF1 and KIF23) from DryNetMC were significantly associated with survival of glioam patients, whereas, in contrast, not all of the top 5 genes from DEseq2 showed prognostic role (**Fig 5E and 5F**). These results demonstrated superior capability of the DryNetMC in discovering potential functional genes compared with the conventional method.

### Temporal pattern similarity of the prioritized genes was predictive of cellular drug sensitivities

**Fig 6:**
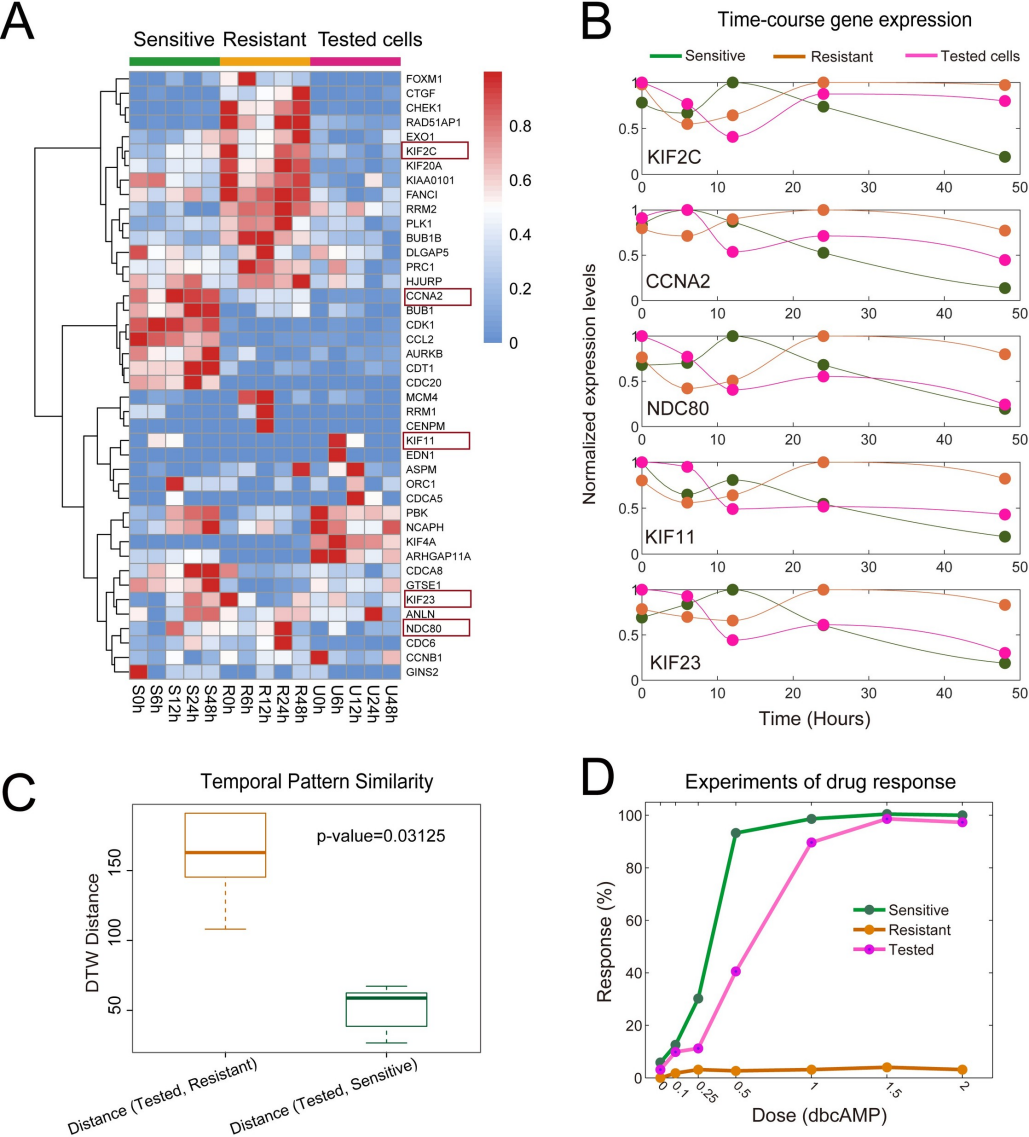
Temporal pattern similarity of the prioritized 5 genes was predictive of drug sensitivity/resistance. RNA-seq data from the U87MG cell line (U) and its response to dbcAMP were used for testing. (**A**) Heat map showing the expression profiles of the genes in the differential network of the sensitive, resistant and tested cells (i.e., U87MG cell line). (**B**) Time-course expression patterns of the 5 genes (i.e., KIF2C, CCNA2, NDC80, KIF11 and KIF23) in the three cell lines. (**C**) The similarity of temporal pattern of the 5 genes between the tested cell line and the sensitive or resistant cell line. Dynamic time warping (DTW) distance was used to measure the temporal expression similarity of the 5 genes between different cellular states. Boxplots showed significant differences in the distributions of pair-wise distances, with p-value equal to 0.03125 (one-tailed Wilcoxon signed rank test), indicating that the tested cell line was more similar to the sensitive cells. (**D**) Experimental data of the dose-response of three cell lines to dbcAMP treatment validated that the response of the tested cell line was much closer to that of the sensitive cells.

We then tested whether the temporal patterns of the prioritized 5 genes (i.e., KIF2C, CCNA2, NDC80, KIF11 and KIF23) could be used to predict drug sensitivities of different glioma cell lines. RNA-seq data from another glioma cell line, U87MG, was used for testing. **Fig 6A** shows the expression profiles of genes in the differential network in the sensitive, resistant and tested cells. **Fig 6B** shows the time-course expression patterns of the prioritized 5 genes in the three cell lines, which were consistent with the qPCR experiments (**S7 Fig**).

We employed the dynamic time warping (DTW) algorithm [37] to measure the temporal pattern similarity between the tested cell line and the sensitive or resistant cell line. **Fig 6C** shows that the DTW distance of the tested cell line to the sensitive cell line was significantly smaller than that to the resistant cell line, with a *p-value* equal to 0.03125 (one-tailed Wilcoxon signed rank test) (see also **S8 Fig**). Therefore, we hypothesized that the response of the tested cells to the cAMP treatment was more similar to that of the sensitive cells. As such, we used our experimental data of the morphological changes exhibited by three cell lines in response to different doses of dbcAMP [1] to validate the drug sensitivity prediction. The percent morphological change of each cell line in response to the dbcAMP treatment was experimentally measured (**Fig 6D**), and the dose-response curve of the tested cell line was closer to that of the sensitive cell line.

**Fig 7:**
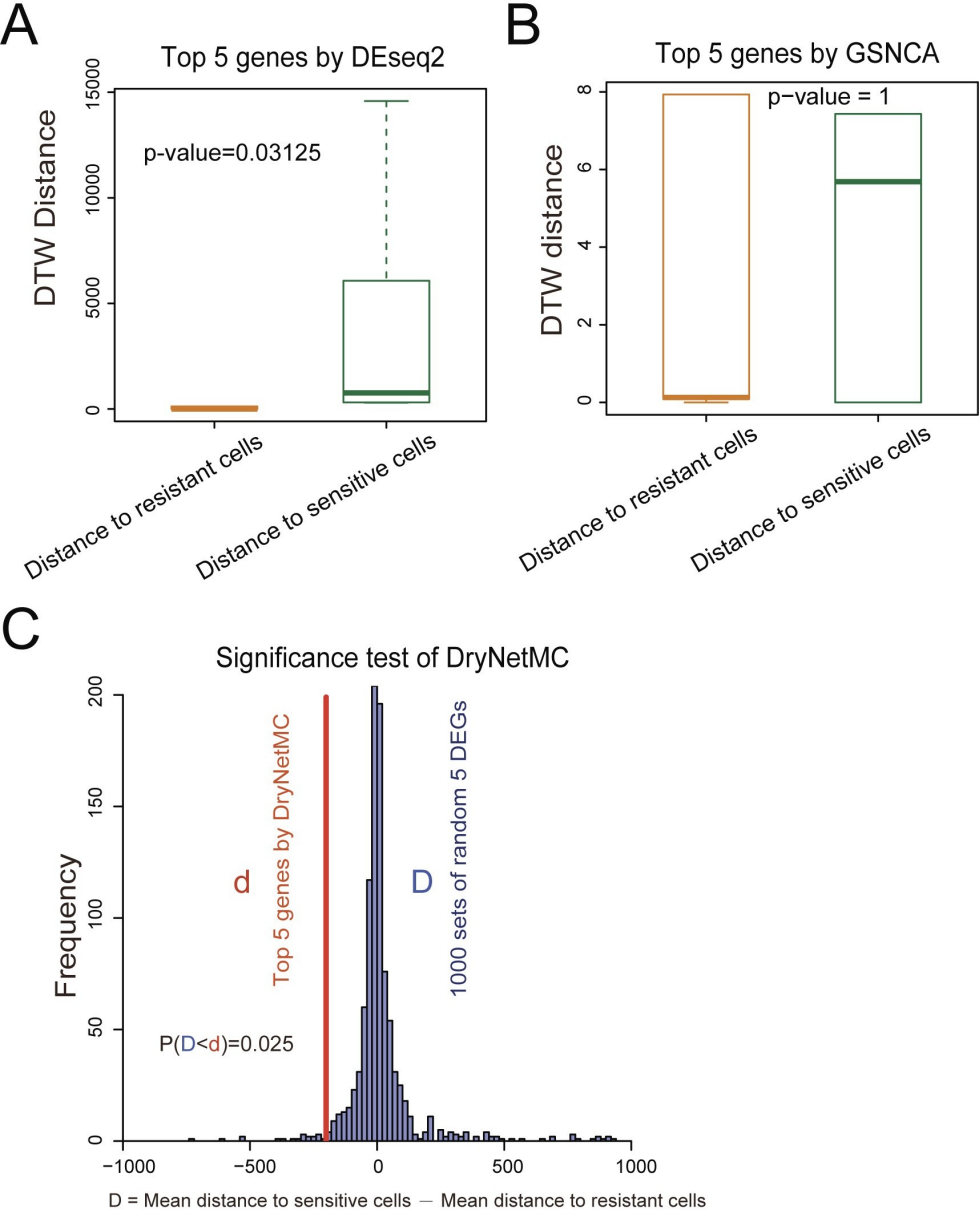
Comparison of the effectiveness of the top 5 genes prioritized by the DryNetMC with that by other methods, including DEseq2 and GSNCA. RNA-seq data of U87MG cells was used to test its distance to sensitive DBTRG-05MG cells or resistant LN-18 cells, based on the expression pattern similarity of the selected genes evaluated using pair-wised DTW distance. (**A-B**) The similarity between the tested cells and the sensitive or resistant cells was evaluated based on the temporal expression patterns of (**A**) the top 5 genes prioritized by DEseq2 (i.e. OCIAD2, FLG, SPP1, SOX3 and NEFL) and (**B**) the top 5 genes prioritized by GSNCA (i.e. C5orf58, COL4A3, MUC16, DKFZp686O1327, GAL). Boxplots show the difference in distance distributions, with the statistical significance evaluated by two-sided Wilcoxon signed rank test. (**C**) The significance test for the top 5 genes prioritized by the DryNetMC using a bootstrapping approach (see details in S1 Text). The resulting p-value was 0.025, indicating non-randomness of the DryNetMC-prioritized 5 genes.

Moreover, we further compared the effectiveness of the top 5 genes prioritized by our method with that by other methods including the conventional differential expression analysis method (e.g., DEseq2 [36]) and the differential co-expression network-based method (e.g., GSNCA [13]). **Fig 7A and 7B** indicate that the expression patterns of the gene set prioritized by DEseq2 (according to fold changes) or by GSNCA (according to high directed preorder traversal of the tree [38]) were not positively correlated with the drug sensitivity. These results demonstrated the distinct functions and advantages of our method in prioritizing key genes responsible for drug resistance based on time-course RNA-seq data.

We further tested the statistical significance of the DryNetMC-prioritized 5 genes using a bootstrapping approach (**S1 Text**). We randomly selected 5 DEGs (analyzed using DEseq2) and set them as marker genes to evaluate the distance from the tested cells to the sensitive cells (*D*_*S*_) or to the resistant cells (*D*_*R*_) (**S9 Fig**). The difference value (*D = D*_*S*_−*D*_*R*_) was calculated. Smaller *D* indicates more closeness of the tested cells to the sensitive cells. The above process was repeated for 1000 times. **Fig 7C** shows the distribution of the above difference values. Let *d* denote the difference value of the two distances derived from the top 5 genes prioritized by the DryNetMC. Probability *P*(*D<d*) was estimated to be 0.025, which justified non-randomness of the DryNetMC-prioritized 5 genes.

## Discussion

Glioma is one of the most malignant primary brain cancers [39], and conventional therapeutics, including surgery, radiotherapy, and chemotherapy (e.g., using drug temozolomide), have resulted in only limited improvement of the median survival of glioma patients [40, 41]. Therefore, more effective drug targets or therapeutic strategies are required. Differentiation therapy using some agents that induce tumor cell differentiation [42], which is different from most chemotherapies that aim to kill cancer cells, has been proposed for the treatment of tumors. However, the molecular mechanisms underlying drug resistance in differentiation therapy remain unclear, which impedes the development and clinical effectiveness of this type of therapy [43, 44].

Elucidating the gene regulatory networks (GRNs) that distinguish the drug-resistant cellular state from the drug-sensitive cellular state is essentially important for gaining systems-level insights, improving our understanding of cancer drug resistance and ultimately developing more effective therapeutic strategies. Due to the development of high-throughput technologies (such as RNA-seq or reverse phase protein arrays (RPPA)), mathematical modeling and computational prediction play increasingly important roles in the study of cancer drug resistance [45, 46]. The present study employed time-course RNA-seq data to model and to characterize the dynamic gene regulatory networks underlying glioma resistance to differentiation therapy. Specifically, we proposed a dynamic network-based computational method to prioritize the key genes responsible for cancer drug resistance, which is conceptually innovative compared to the conventional differential expression method (e.g., DEseq2). In addition, our method is verified to be more accurate for inferring GRNs compared to the other methods. We applied our computational method to realistic RNA-seq dataset of gliomas, and several novel predictions were verified by the additional gene expression/cellular response data or the clinical data. Our study provides a principled approach to gain insights into dynamic adaptation and regulatory network mechanisms of cancer drug resistance, and sheds lights on designing new biomarkers or targets for predicting or overcoming drug resistance.

Our study led to several novel and interesting biological discoveries, which were verified by cell line or clinical data or supported by the literature, as described below.

First, we found that gene expression in the sensitive and resistant cells showed different temporal patterns in response to cAMP treatment (**Fig 2**). The temporally changing genes in the sensitive cells exhibited more monotone expression patterns, whereas the resistant cells showed more adaptive gene expression patterns. Moreover, the temporal patterns of the genes in the differential network were associated with drug sensitivity or resistance, which was verified by *in vitro* experimental data (**Fig 6**). In this sense, the temporal pattern could be used as a biomarker for predicting the sensitivity or resistance of different glioma cells to a drug used in differentiation therapy.

Second, the reconstructed sensitive network and resistant network were found to exhibit significantly differences with respect to network topology, local entropy and gene expression dynamics (**Fig 4**). We thus proposed a novel index to comprehensively measure node importance. Based on the node importance score, we developed a differential regulatory network-based biomarker model to identify the most influential genes in the differential network for predicting and controlling drug resistance. The clinical data verified the association of the identified genes (i.e., KIF2C, CCNA2, NDC80, KIF11, KIF23, ANLN, and CENPM) with the targeted therapy response and prognosis of glioma patients (**Fig 5**).

Third, the identified genes are all key regulators in cell cycle pathways that were revealed to play important roles in glioma differentiation and drug resistance (**Fig 3**). The roles of cell cycle have been implicated in drug resistance for more than two decades [47]. Furthermore, the cell cycle position of single cancer cells is related to the sensitivity to the chemotherapeutic drugs [48]. Previous experimental studies have demonstrated that changes in the expression of several cell cycle regulators were accompanied by cAMP-dependent differentiation in C6 glioma [49]. These experimental evidences supported our predictions that the cell cycle plays important roles in glioma differentiation and drug resistance during differentiation therapy. In addition, in our previous studies, we developed a mechanism-based mechanistic modeling approach for studying signaling networks underlying cancer drug resistance [50–53]. The cell cycle regulator cyclin D1 was mathematically revealed and experimentally validated to control glioma differentiation, which is consistent with the discovery in this study. It would be valuable to further test whether inhibiting cell cycle could induce glioma cell differentiation in the future work.

In future studies, we will use single-cell RNA-seq data to analyze the heterogeneity of tumor cells [54], and we will thus infer the pseudo-trajectory of glioma differentiation and construct the corresponding gene regulatory network using the approach developed in this study. Using this approach, we will compare the evolutionary trajectories of drug-sensitive and drug-resistant cells and analyze the difference between the two gene regulatory networks.

In summary, our study developed a time-course RNA-seq data-driven computational method to study the network mechanisms underlying cancer drug resistance. Several novel predictions derived from realistic biological data were verified by *in vitro* experimental or clinical data. The proposed approach provides a strategy to gain insights into the dynamic adaptation and regulatory network mechanisms of cancer drug resistance and sheds light on the design of novel biomarkers or targets for predicting or overcoming drug resistance.

## Supporting information

S1 Fig Differential sensitivities of DBTRG and LN18 cell lines in response to the treatment of dbcAMP. **(A-B)** Morphological changes of DBTRG and LN18 cell lines following the treatment of dbcAMP for 48 hr. DBTRG-05MG cells showed significant morphological change, while LN-18 cells did not. **(C-D)** Western blots of GFAP in DBTRG (C) and LN18 (D) cell lines, respectively, treated with dbcAMP. GFAP is a marker for glioma cell differentiation. (**E-F**) Quantification of GFAP activities in two cell lines, normalized to levels of tublin. GFAP increased significantly in DBTRG cell line, but not in LN-18 cell line, after treatment of dbcAMP for 48 hr. These data demonstrated that, induced by the treatment of cAMP activator, DBTRG-05MG cells differentiated to glio-like cells, while LN-18 cells did not.
(TIF)

S2 Fig Quantifying and comparing monotonic changes or adaptive changes of the TCGs in sensitive and resistant cells. (**A-B**) Illustration showing the definition of scores for monotonic response and adaptive response. (**C**) Comparison of monotonic responses of TCGs between the sensitive cells and the resistant cells. (**D**) Comparison of adaptive responses of TCGs between the sensitive cells and the resistant cells. One-tailed Wilcoxon rank sum test p-values were used to assess the statistical significance. These results indicated that the TCGs in the resistant cells tended to have higher adaptive response scores but lower monotonic response scores compared to the TCGs in the sensitive cells.
(TIF)

S3 FigIllustration and validation of the DryNetMC for network inference based on a simulated dataset.(**A**) A true network with typical motifs, such as positive and negative feedback loops and crosstalk. A system of ODEs, in the form of $\frac{{dx}_{i}}{dt}=\sum_{i=1}^{5}a_{ij}x_{j}+b_{i}$, (*i* = 1,2,…,5), was built to generate the original time course gene expression data. The interaction confidents (*a*_*ij*_) were given in (**B**) and the degradation rates (*b*_*ij*_) were set to -0.1. The original simulated data, the sampling data mimicking the experimental measurements (at 0, 6, 12, 24, 48 hr) and the Hermit interpolations were shown in (**C**), where a.u. denotes arbitrary units. (**D**) ROC curves comparing the accuracies of the DryNetMC with other methods with respect to predicting the occurrence of the true network edges. The methods used for comparison include PCC-based correlation network method (PCCNet), tree-based ensemble learning methods (GENIE3), the state-of-the-art ODE-LASSO method (OdeLasso), the method incorporating prior information (OdeLassoP) and a dynamic Bayesian network method (GRENITS). AUC of the DryNetMC (0.7937) is much greater than that of other methods.(TIF)

S4 Fig Comparison of the network inference accuracy of DryNetMC with other methods based on 100 simulated datasets. 100 true networks were synthetically generated (see details in **S1 Text**). The methods used for comparison include PCC-based correlation network method (PCCNet), tree-based ensemble learning methods (GENIE3), ODE-LASSO method (OdeLasso), the ODE-LASSO method incorporating prior information (OdeLassoP) and a dynamic Bayesian network method (GRENITS). The DryNetMC significantly outperformed other methods. One-tailed Wilcoxon signed rank test p-values are 2.849101e-16 (for DryNetMC vs. PCCNet), 8.688378e-17 (for DryNetMC vs. GENIE3), 3.84935e-15 (for DryNetMC vs. GRENITS), 4.071108e-18 (for DryNetMC vs. OdeLasso) and 7.868774e-12 (for DryNetMC vs. OdeLassoP).
(TIF)

S5 Fig Parameter estimation and edge selection in the network models. (**A-B**) The frequency distribution of penalty weights in the LASSO regressions for sensitive network and resistant network, respectively. (**C-D**) The frequency distribution of mean cross-validation errors for sensitive network and resistant network, respectively. (**E-F**) Significant edge selection using BIC for sensitive network and resistant network, respectively. The minimal absolute values of edge strength (θ) was achieved around 0.01.
(TIF)

S6 Fig Edge number changes against the interaction strength. (TIF)

S7 Fig qPCR validation of temporal expressions of 5 top ranked marker genes in both DBTRG and LN18 cell lines fallowing the treatment of dbcAMP for 48 hr. (**A, C, E, G, I**) DBTRG cell line. (**B, D, F, H, J**) LN-18 cell line. DBTRG cells (i.e., the sensitive cells) and LN-18 cells (i.e., the resistant cells) showed distinct temporal expression profiles of the identified genes (i.e., KIF2C, CCNA2, NDC80, KIF11, and KIF23). The expressions of these genes decreased dramatically in DBTRG cells after the dbcAMP treatment for 48 hr, while (adaptively) increased in LN-18 cells. Expression patterns were similar to what was found in the RNA-seq data. The raw data of the qPCR were provided in online supplementary file (Raw data of qPCR experiments.rar).
(TIF)

S8 Fig Temporal pattern similarity evaluated using Euclid distance or Manhattan distance. The distance from the tested cell line to the sensitive cell line or to the resistant cell line was evaluated based on the temporal pattern similarity of the DryNetMC-derived 5 genes (i.e., KIF2C, CCNA2, NDC80, KIF11, and KIF23) by using (**A**) Euclid distance or (**B**) Manhattan distance. RNA-seq data of U87MG cells was used to calculate its distance to sensitive DBTRG-05MG cells or resistant LN-18 cells, by calculating the pair-wised distance of the 5 genes. Boxplots show the significant difference in distance distributions. The statistical significance was assessed using one-tailed Wilcoxon signed rank test.
(TIF)

S9 Fig The distances between the tested cells and the sensitive or resistant cells evaluated based on the temporal expression patterns of 1000 sets of randomly selected 5 DEGs. RNA-seq data of U87MG cells was used to test its distance to sensitive DBTRG-05MG cells or resistant LN-18 cells, based on the expression pattern similarity of the selected genes evaluated using pair-wised DTW distance. Boxplot shows the mean distance to the resistant cells (green) and mean distance to the sensitive cells (purple), respectively. Two-sided Wilcoxon signed rank test p-value (0.9292) was used to assess the statistical significance of the difference in distance distributions.
(TIF)

S1 Data Raw data of qPCR experiments. (RAR)

S1 Table Measuring the importance of each node in the differential network. The first 4 columns list the gene names, hub score, entropy and adaptation score of each node. The following columns 5–7 list the rank of hub score, entropy and adaptation score for the corresponding nodes. The column 8 lists the scores of node importance, and the last column ranks each node accordingly.
(TIF)

S2 Table The annotation of genes in the differential network with their rankings. The symbol, description and ranking for each gene was listed.
(TIF)

S1 Text Supplementary computational methods. The file includes the following sections: RNA-seq data processing, Data interpolation, Construction of the initial correlation network, Validation and comparison of the DryNetMC with other methods, Identifying DryNB for predicting drug-resistance of glioma patients, Assessing the association of the DryNB genes with prognosis of glioma patients, Significance test of the prioritized genes, and Supplementary references.
(PDF)

S2 Text Supplementary experimental methods. The file includes the following sections: Cell culture and reagents, Western Blotting, qPCR and Morphological imaging.
(PDF)
