# Supplementary figures and images for: Differential regulatory network-based quantification and prioritization of key genes underlying cancer drug resistance based on time-course RNA-seq data
Source: PLoS Comput Biol. 2019 Nov 4;15(11):e1007435. doi: 10.1371/journal.pcbi.1007435 (PMC6827891; doi:10.1371/journal.pcbi.1007435)

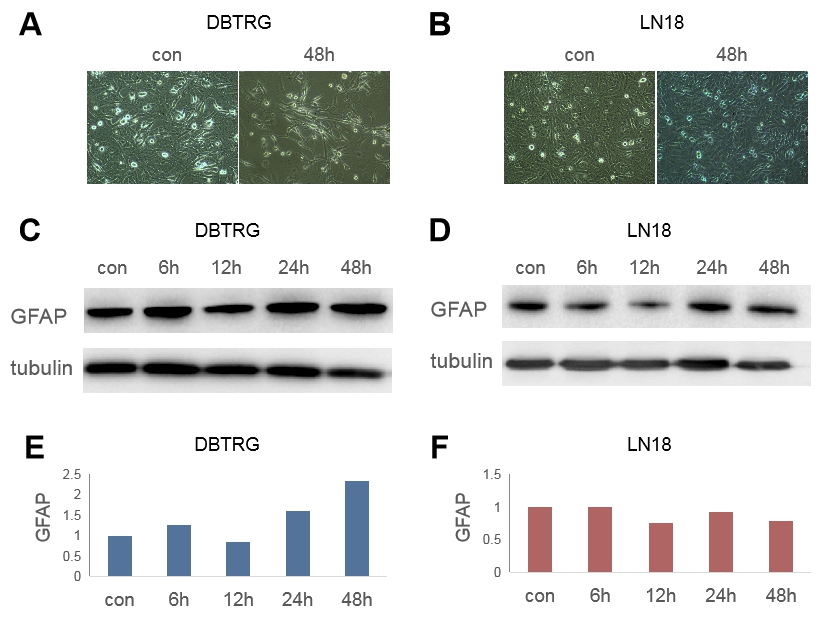

Supplement: S1 Fig — (A-B) Morphological changes of DBTRG and LN18 cell lines following the treatment of dbcAMP for 48 hr. DBTRG-05MG cells showed significant morphological change, while LN-18 cells did not. (C-D) Western blots of GFAP in DBTRG (C) and LN18 (D) cell lines, respectively, treated with dbcAMP. GFAP is a marker for glioma cell differentiation. (E-F) Quantification of GFAP activities in two cell lines, normalized to levels of tublin. GFAP increased significantly in DBTRG cell line, but not in LN-18 cell line, after treatment of dbcAMP for 48 hr. These data demonstrated that, induced by the treatment of cAMP activator, DBTRG-05MG cells differentiated to glio-like cells, while LN-18 cells did not. (TIF) [file pcbi.1007435.s001.tif]

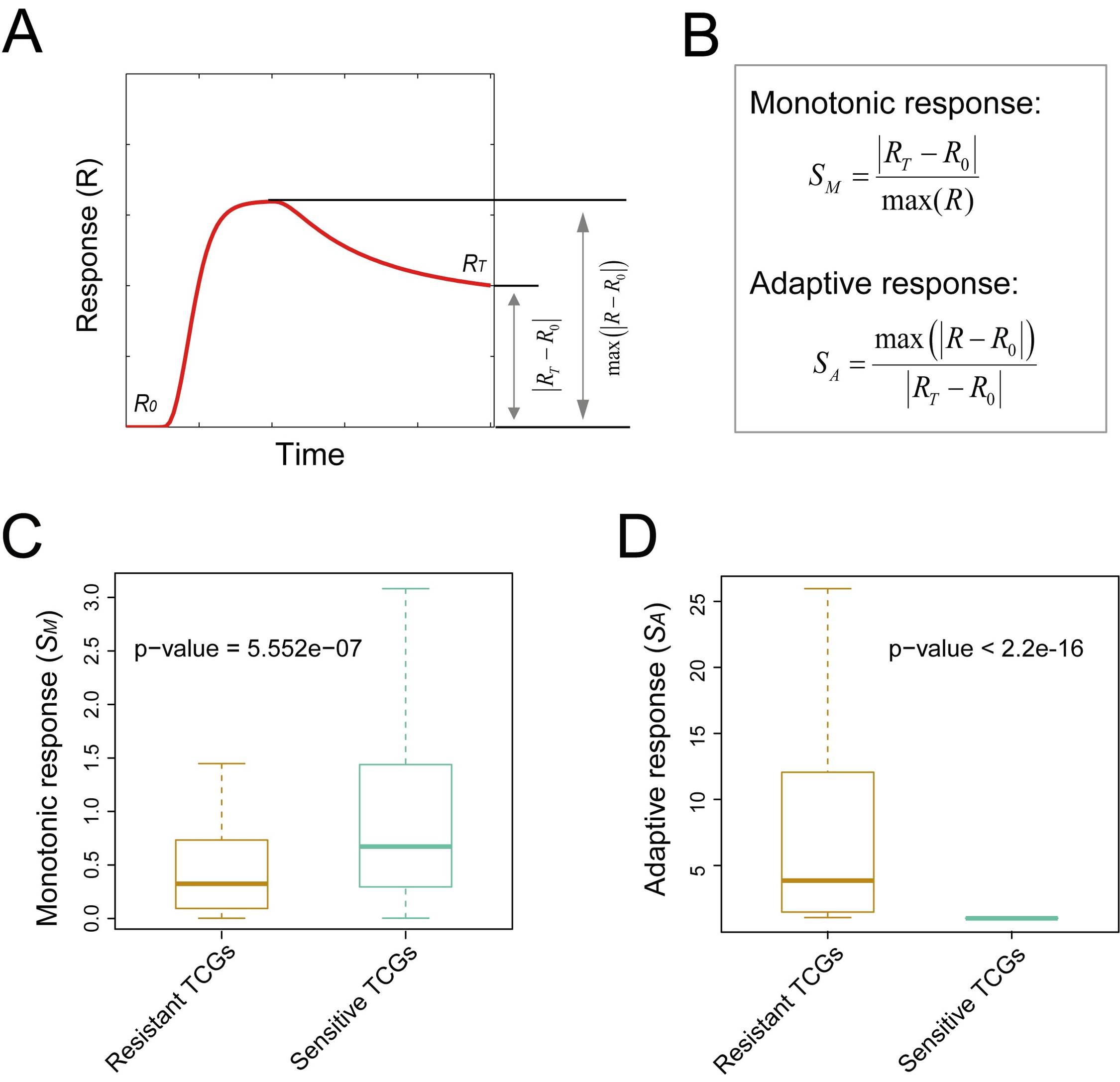

Supplement: S2 Fig — (A-B) Illustration showing the definition of scores for monotonic response and adaptive response. (C) Comparison of monotonic responses of TCGs between the sensitive cells and the resistant cells. (D) Comparison of adaptive responses of TCGs between the sensitive cells and the resistant cells. One-tailed Wilcoxon rank sum test p-values were used to assess the statistical significance. These results indicated that the TCGs in the resistant cells tended to have higher adaptive response scores but lower monotonic response scores compared to the TCGs in the sensitive cells. (TIF) [file pcbi.1007435.s002.tif]

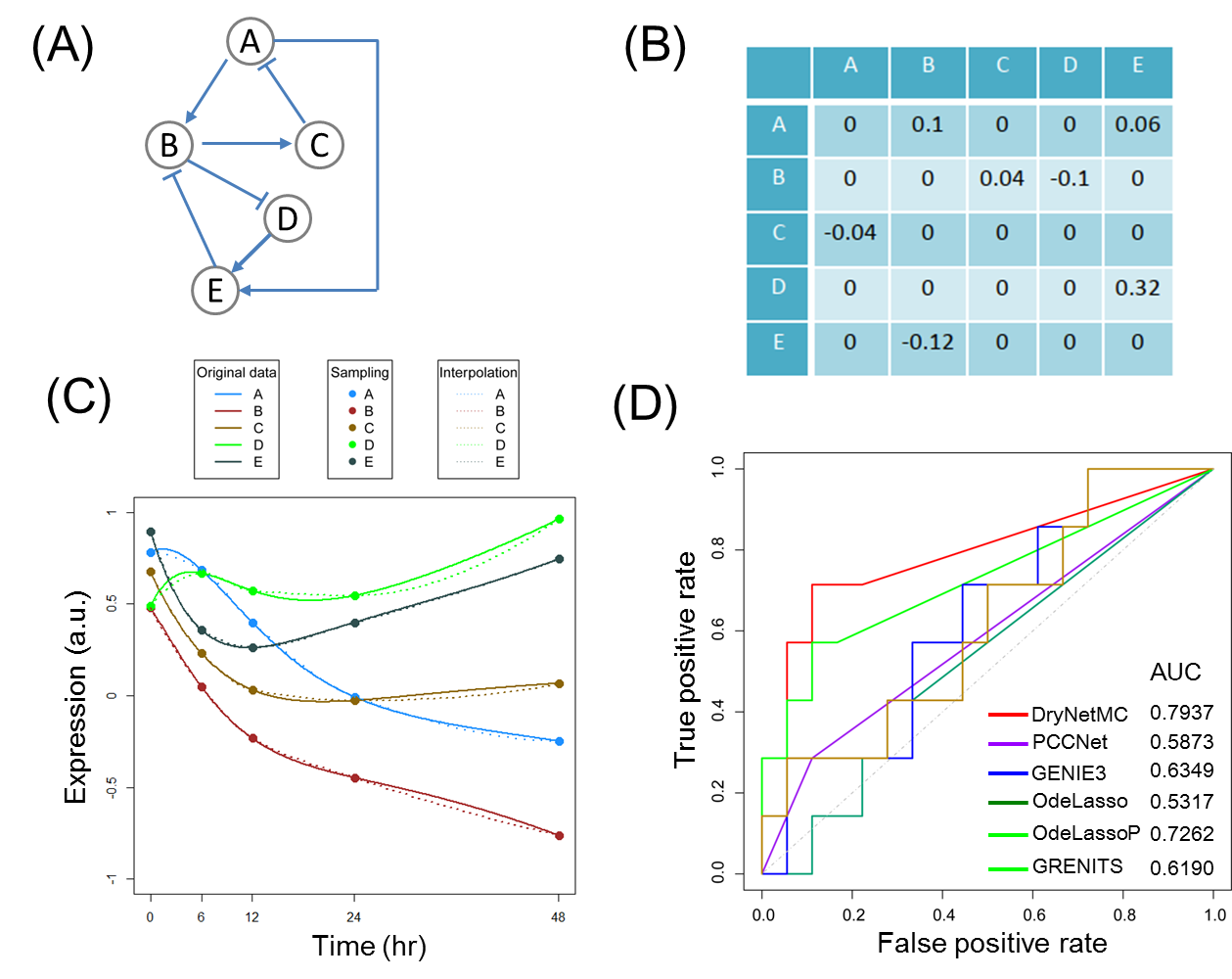

Supplement: S3 Fig — (A) A true network with typical motifs, such as positive and negative feedback loops and crosstalk. A system of ODEs, in the form of dxidt=∑i=15aijxj+bi, (i = 1,2,…,5), was built to generate the original time course gene expression data. The interaction confidents (aij) were given in (B) and the degradation rates (bij) were set to -0.1. The original simulated data, the sampling data mimicking the experimental measurements (at 0, 6, 12, 24, 48 hr) and the Hermit interpolations were shown in (C), where a.u. denotes arbitrary units. (D) ROC curves comparing the accuracies of the DryNetMC with other methods with respect to predicting the occurrence of the true network edges. The methods used for comparison include PCC-based correlation network method (PCCNet), tree-based ensemble learning methods (GENIE3), the state-of-the-art ODE-LASSO method (OdeLasso), the method incorporating prior information (OdeLassoP) and a dynamic Bayesian network method (GRENITS). AUC of the DryNetMC (0.7937) is much greater than that of other methods. (TIF) [file pcbi.1007435.s003.tif]

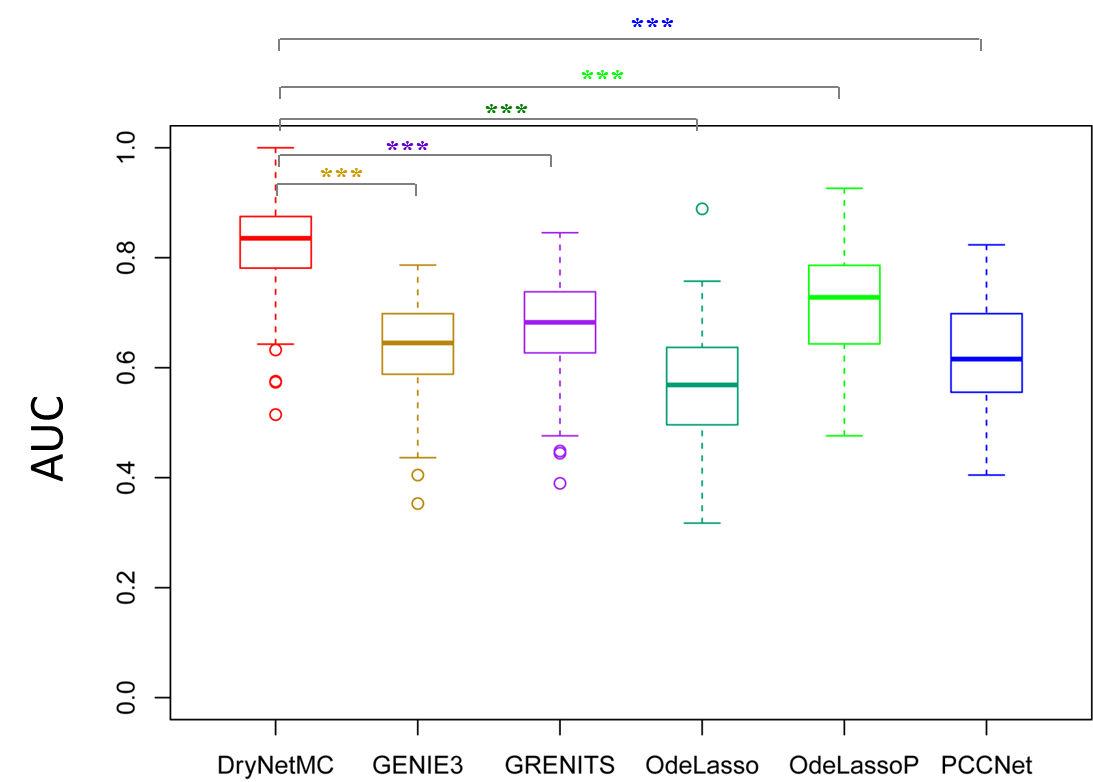

Supplement: S4 Fig — 100 true networks were synthetically generated (see details in S1 Text). The methods used for comparison include PCC-based correlation network method (PCCNet), tree-based ensemble learning methods (GENIE3), ODE-LASSO method (OdeLasso), the ODE-LASSO method incorporating prior information (OdeLassoP) and a dynamic Bayesian network method (GRENITS). The DryNetMC significantly outperformed other methods. One-tailed Wilcoxon signed rank test p-values are 2.849101e-16 (for DryNetMC vs. PCCNet), 8.688378e-17 (for DryNetMC vs. GENIE3), 3.84935e-15 (for DryNetMC vs. GRENITS), 4.071108e-18 (for DryNetMC vs. OdeLasso) and 7.868774e-12 (for DryNetMC vs. OdeLassoP). (TIF) [file pcbi.1007435.s004.tif]

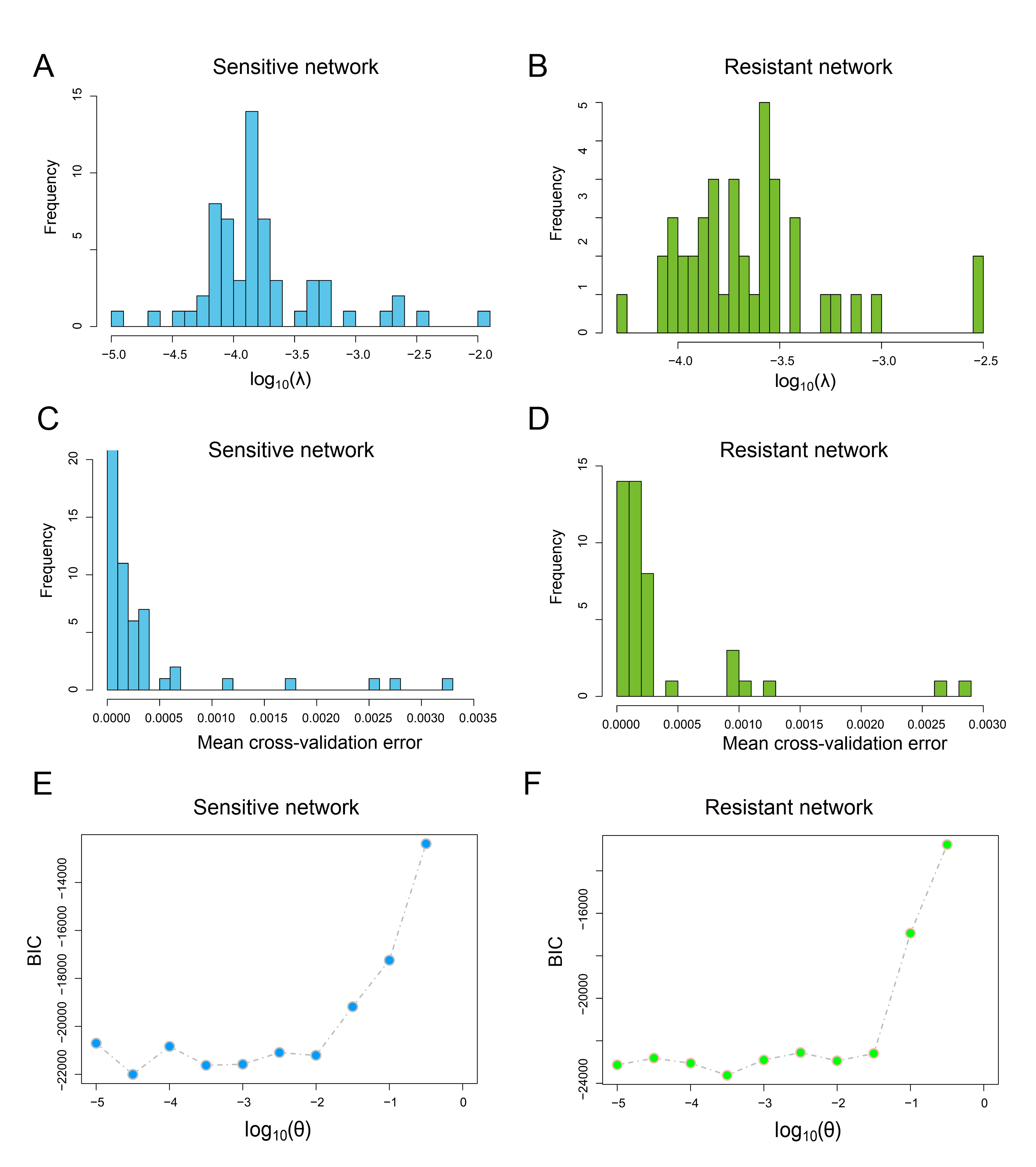

Supplement: S5 Fig — (A-B) The frequency distribution of penalty weights in the LASSO regressions for sensitive network and resistant network, respectively. (C-D) The frequency distribution of mean cross-validation errors for sensitive network and resistant network, respectively. (E-F) Significant edge selection using BIC for sensitive network and resistant network, respectively. The minimal absolute values of edge strength (θ) was achieved around 0.01. (TIF) [file pcbi.1007435.s005.tif]

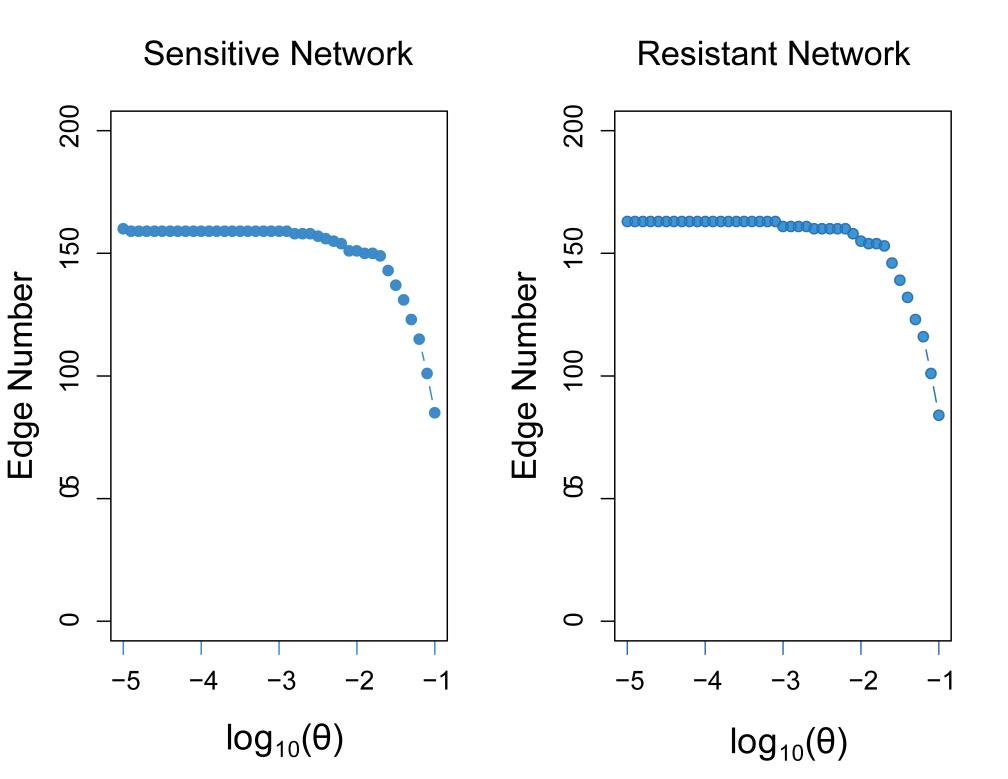

Supplement: S6 Fig — (TIF) [file pcbi.1007435.s006.tif]

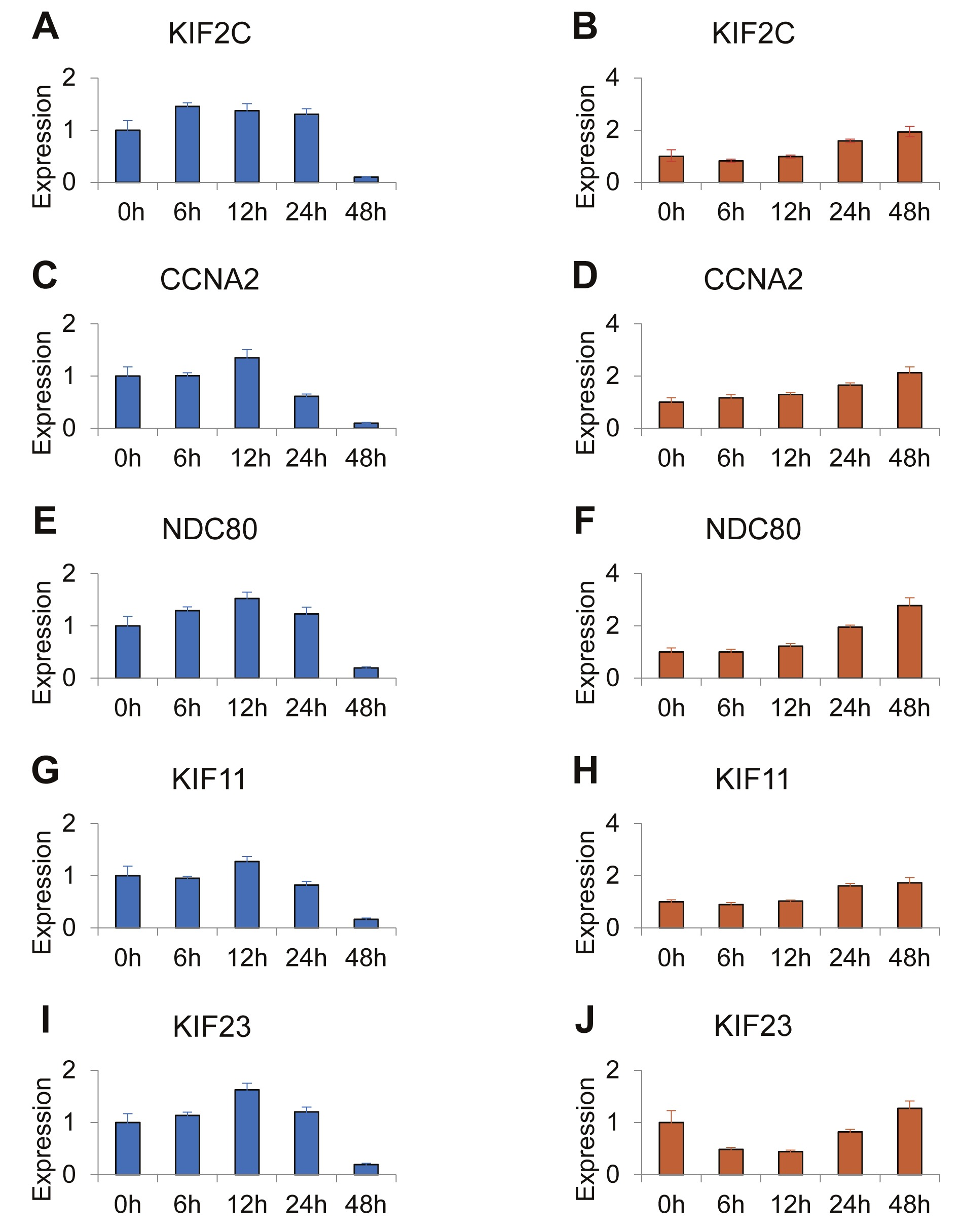

Supplement: S7 Fig — (A, C, E, G, I) DBTRG cell line. (B, D, F, H, J) LN-18 cell line. DBTRG cells (i.e., the sensitive cells) and LN-18 cells (i.e., the resistant cells) showed distinct temporal expression profiles of the identified genes (i.e., KIF2C, CCNA2, NDC80, KIF11, and KIF23). The expressions of these genes decreased dramatically in DBTRG cells after the dbcAMP treatment for 48 hr, while (adaptively) increased in LN-18 cells. Expression patterns were similar to what was found in the RNA-seq data. The raw data of the qPCR were provided in online supplementary file (Raw data of qPCR experiments.rar). (TIF) [file pcbi.1007435.s007.tif]

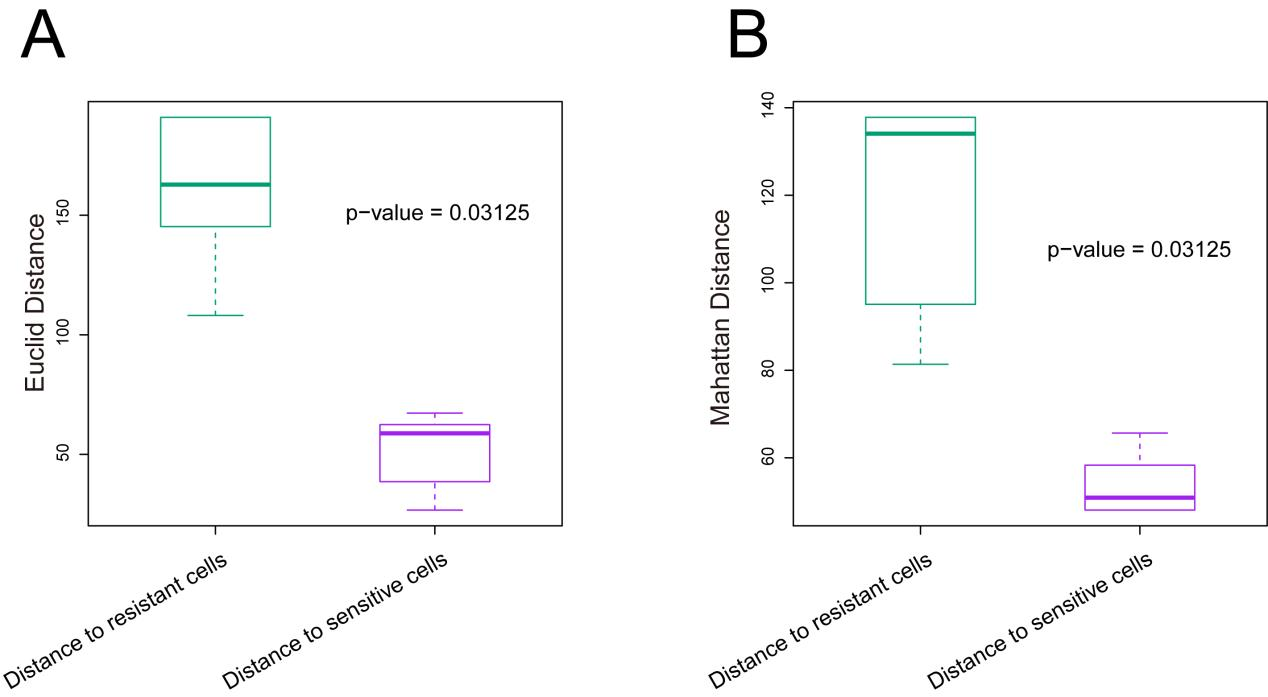

Supplement: S8 Fig — The distance from the tested cell line to the sensitive cell line or to the resistant cell line was evaluated based on the temporal pattern similarity of the DryNetMC-derived 5 genes (i.e., KIF2C, CCNA2, NDC80, KIF11, and KIF23) by using (A) Euclid distance or (B) Manhattan distance. RNA-seq data of U87MG cells was used to calculate its distance to sensitive DBTRG-05MG cells or resistant LN-18 cells, by calculating the pair-wised distance of the 5 genes. Boxplots show the significant difference in distance distributions. The statistical significance was assessed using one-tailed Wilcoxon signed rank test. (TIF) [file pcbi.1007435.s008.tif]

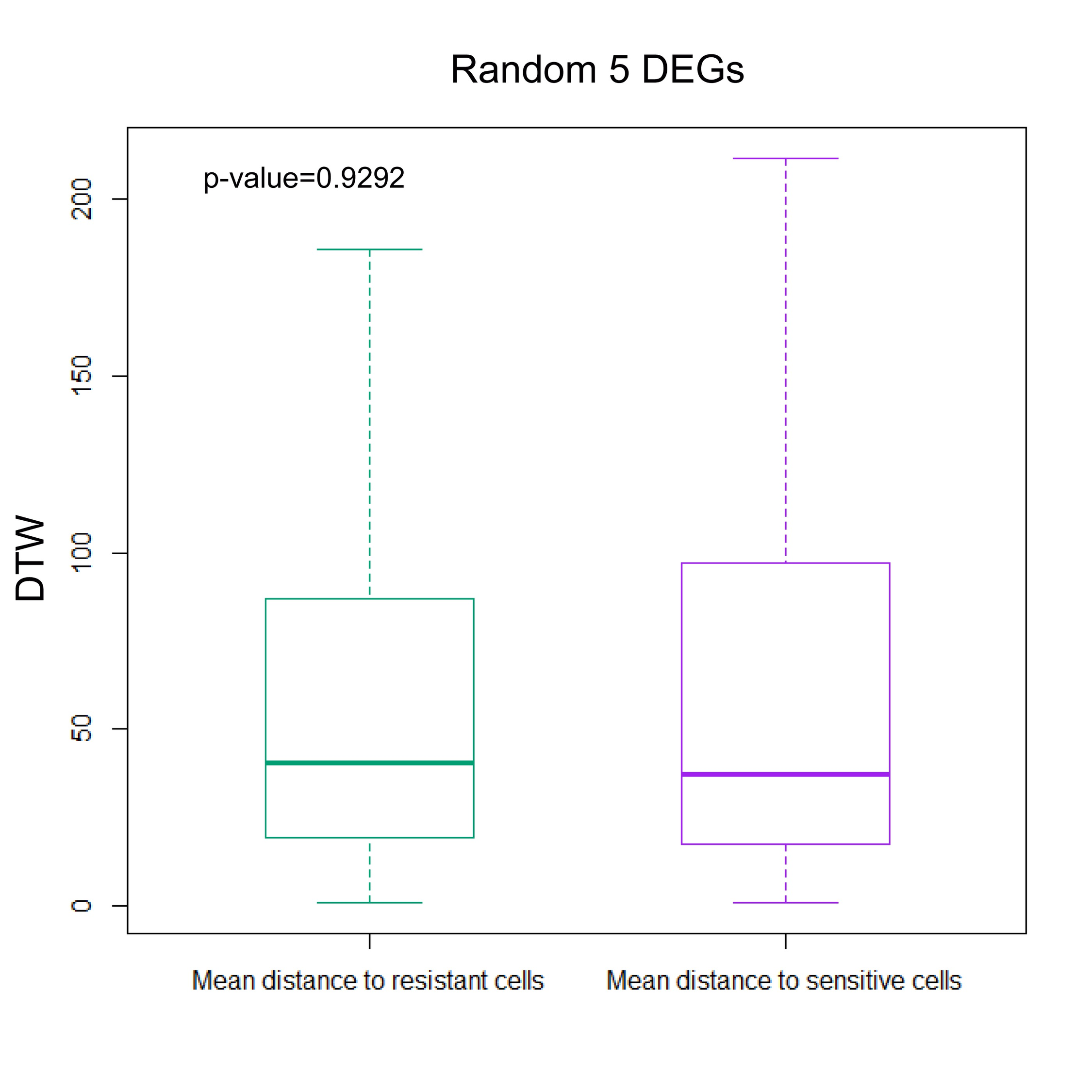

Supplement: S9 Fig — RNA-seq data of U87MG cells was used to test its distance to sensitive DBTRG-05MG cells or resistant LN-18 cells, based on the expression pattern similarity of the selected genes evaluated using pair-wised DTW distance. Boxplot shows the mean distance to the resistant cells (green) and mean distance to the sensitive cells (purple), respectively. Two-sided Wilcoxon signed rank test p-value (0.9292) was used to assess the statistical significance of the difference in distance distributions. (TIF) [file pcbi.1007435.s009.tif]

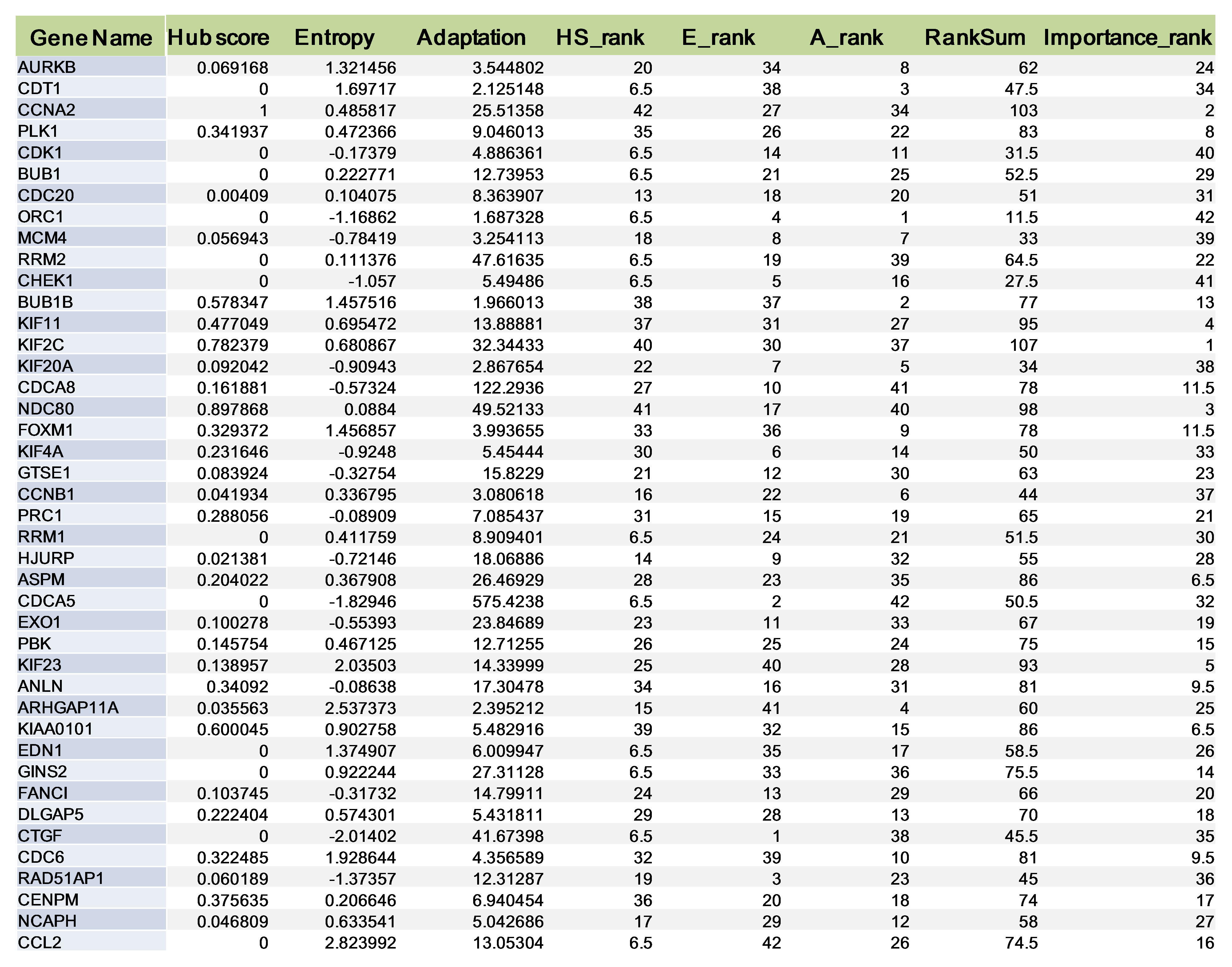

Supplement: S1 Table — The first 4 columns list the gene names, hub score, entropy and adaptation score of each node. The following columns 5–7 list the rank of hub score, entropy and adaptation score for the corresponding nodes. The column 8 lists the scores of node importance, and the last column ranks each node accordingly. (TIF) [file pcbi.1007435.s011.tif]

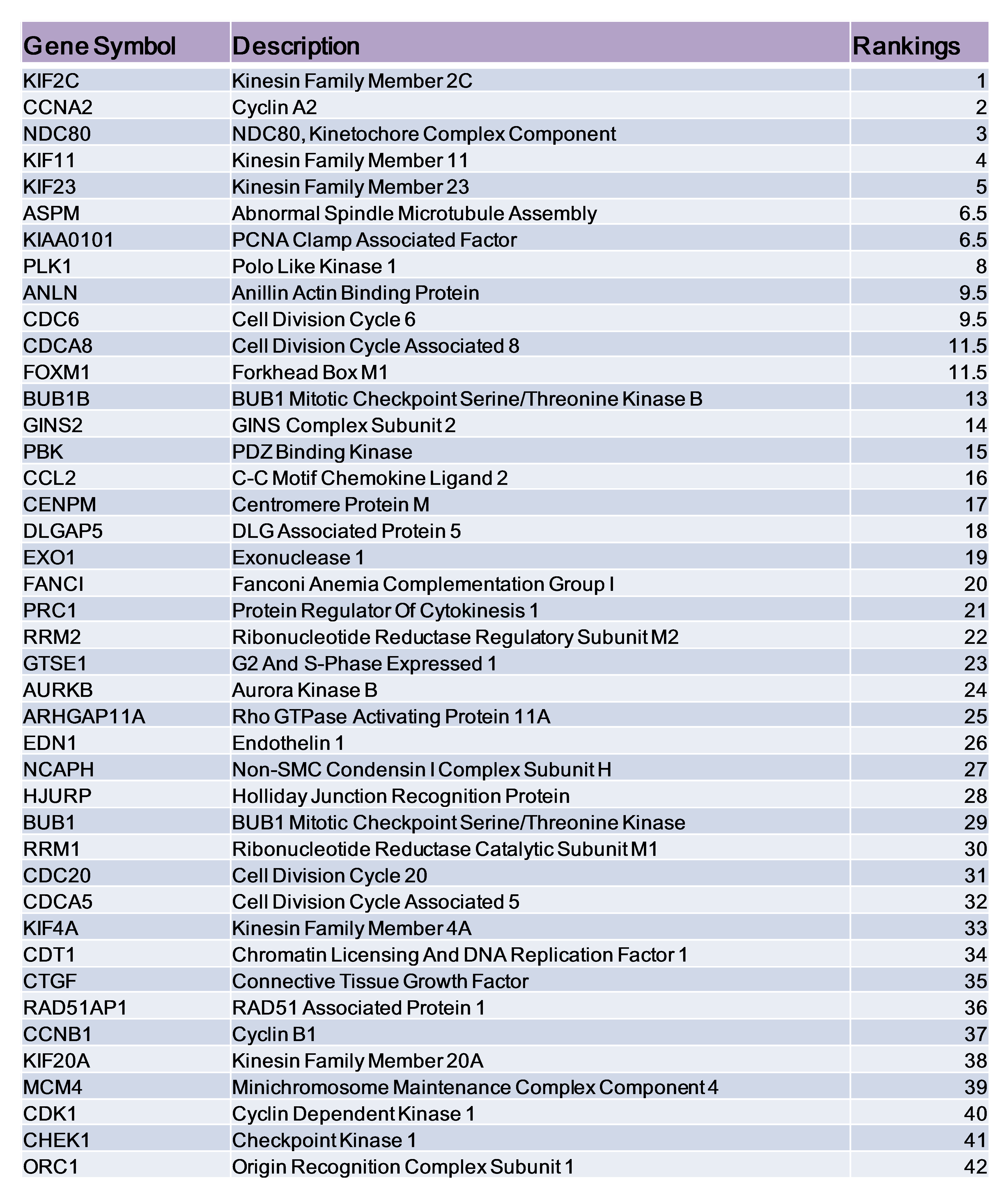

Supplement: S2 Table — The symbol, description and ranking for each gene was listed. (TIF) [file pcbi.1007435.s012.tif]
