# Supplementary material for: Differential regulatory network-based quantification and prioritization of key genes underlying cancer drug resistance based on time-course RNA-seq data
Source: PLoS Comput Biol. 2019 Nov 4;15(11):e1007435. doi: 10.1371/journal.pcbi.1007435 (PMC6827891; doi:10.1371/journal.pcbi.1007435)
Supplement: S1 Text — The file includes the following sections: RNA-seq data processing, Data interpolation, Construction of the initial correlation network, Validation and comparison of the DryNetMC with other methods, Identifying DryNB for predicting drug-resistance of glioma patients, Assessing the association of the DryNB genes with prognosis of glioma patients, Significance test of the prioritized genes, and Supplementary references. (PDF) [file pcbi.1007435.s013.pdf]

Supplementary Materials for

Differential regulatory network-based quantification and prioritization of key genes underlying cancer drug resistance based on time-course RNA-seq data

Jiajun Zhang[1#], Wenbo Zhu[2#], Qianliang Wang[1], Jiayu Gu[2], L. Frank Huang[3,4*], Xiaoqiang Sun[5,1*]

[1] School of Mathematics, Sun Yat-Sen University, Guangzhou 510080, China.

[2] Department of Pharmacology, Zhongshan School of Medicine, Sun Yat-Sen University, Guangzhou 510080, China.

[3] Brain Tumor Center, Division of Experimental Hematology and Cancer Biology, Cincinnati Children's Hospital Medical Center, Cincinnati, OH 45229, USA.

[4] Department of Pediatrics, University of Cincinnati College of Medicine, Cincinnati, OH 45229, USA.

[5] Department of Medical Informatics, Zhongshan School of Medicine, Sun Yat-Sen University, Guangzhou 510080, China; Key Laboratory of Tropical Disease Control (Sun Yat-Sen University), Chinese Ministry of Education, Guangzhou 510080, Guangdong, China.

* To whom correspondence should be addressed. Tel: 86-020-87330128; Fax: 86-020-87330420; Email: sunxq6@mail.sysu.edu.cn or xiaoqiangsun88@gmail.com (XS; Lead contact); Frank.Huang@cchmc.org or hleicug@gmail.com (LH)

Text S1 Supplementary computational methods

RNA-seq data processing

The raw reads were processed using a standard pipeline as follows: We firstly filtered the low quality tags and trimmed the raw reads to 90 nucleotides (nt) of length. Next we used TopHat [1] (http://tophat.cbcb.umd.edu/) to map the clean reads to the UBSC human genome browser database (UCSC) hg19 reference genome [2] (http://genome.ucsc.edu/). We then applied the HTSeq program [3] and DESeq package [4] to calculate the expression levels of transcripts and to perform differential expression analysis, respectively.

Data interpolation

Given that the total number of time points of RNA-seq data in practice is often relatively small, we sampled more data points of the TCG expression data using a Hermit polynomial interpolation method [5]. The piecewise cubic Hermit interpolation polynomial $x(t)$ was constructed to approximate the gene expression level u_i (i=0, 1,..., K) on each subinterval [T_i, T_{i+1}] (i=0, 1,..., K-1), such that $x(T_i)=u_i$ and $x'(T_i)=u_i'$, where u_i' is the specified value of changing rate of the original data at T_i.

The piecewise cubic Hermit interpolation polynomial $x(t)$ has the following form:

$$x(t)=\sum_{i=0}^{K}\left(u_i h_i(t)+u_i' H_i(t)\right) \tag{S1}$$

where $h_i(t)$ and $H_i(t)$ are interpolation cardinal functions and they have the follow formulas:

$$h_0(t)=\begin{cases}\left(1+2\dfrac{t-T_0}{T_1-T_0}\right)\left(\dfrac{t-T_1}{T_0-T_1}\right)^2 & T_0\le t\le T_1\\ 0 & T_1\le t\le T_K\end{cases} \tag{S2}$$

$$h_i(t)=\begin{cases}\left(1+2\dfrac{t-T_0}{T_{i-1}-T_i}\right)\left(\dfrac{t-T_{i-1}}{T_i-T_{i-1}}\right)^2 & T_{i-1}\le t\le T_i\\ \left(1+2\dfrac{t-T_0}{T_{i+1}-T_i}\right)\left(\dfrac{t-T_{i+1}}{T_i-T_{i+1}}\right)^2 & T_i\le t\le T_{i+1}\\ 0 & [T_0,T_K]\backslash[T_{i-1},T_{i+1}]\end{cases} \tag{S3}$$

$i=1,2,\cdots,n-1$

$$h_n(t)=\begin{cases}\left(1+2\dfrac{t-T_K}{T_{K-1}-T_K}\right)\left(\dfrac{t-T_{K-1}}{T_K-T_{K-1}}\right)^2 & T_{K-1}\le t\le T_K\\ 0 & T_0\le t\le T_{K-1}\end{cases} \tag{S4}$$

$$H_0(t)=\begin{cases}\left(t-T_0\right)\left(\dfrac{t-T_1}{T_0-T_1}\right)^2 & T_0\le t\le T_1\\ 0 & T_1\le t\le T_K\end{cases} \tag{S5}$$

$$H_i(t)=\begin{cases}\left(t-T_i\right)\left(\dfrac{t-T_{i-1}}{T_i-T_{i-1}}\right)^2 & T_{i-1}\le t\le T_i\\ \left(t-T_i\right)\left(\dfrac{t-T_{i+1}}{T_i-T_{i+1}}\right)^2 & T_i\le t\le T_{i+1}\\ 0 & [T_0,T_K]\backslash[T_{i-1},T_{i+1}]\end{cases} \tag{S6}$$

$$i=1,2,\cdots,n-1$$

$$H_n(t)=\begin{cases}\left(t-T_K\right)\left(\dfrac{t-T_{K-1}}{T_K-T_{K-1}}\right)^2 & T_{K-1}\le t\le T_K\\ 0 & T_0\le t\le T_{K-1}\end{cases} \tag{S7}$$

The piecewise cubic Hermit interpolation used here preserves the monotonicity, local extremum and nonnegativity of the expression data. We noted that other interpolation methods, such as polynomial spline or cubic spline, might result in unrealistic negative interpolated values and unexpected variations in gene expression.

We then uniformly took n (for example, n=48 or 100) data points from $x(t)$, denoting them as $x(t_1)$, $x(t_2)$, $\cdots x(t_n)$. Below we show that the data interpolation in the DryNetMC improves the network inference accuracy.

Construction of the initial correlation network

Prior knowledge of functional interactions among the selected TCGs was used to construct an initial gene network. Specifically, an initial gene network of the TCGs was built using the STRING database [6-8] (https://string-db.org/), which integrates known and predicted functional associations between proteins/genes derived from genomic context, high-throughput experiments, conserved coexpression studies, the published literature or previous knowledge. In this study, a ‘high’ confidence level (0.7) in the database was set for the construction of the initial network. The network connection information, including direct physical interactions and indirect functional associations, was extracted and represented as a graph $G(V, E)$.

We then calculated the Pearson correlation coefficient (PCC) between each pair of nodes in the above graph using the time-course gene expression data generated from the RNA-seq transcriptome. Correlation networks were built for both sensitive cells and resistant cells. An edge in $G(V, E)$ was selected as a significant correlation edge if the p-value of the correlation was less than 0.05, and the other edges were considered non-significant correlations and deleted from $G(V, E)$.

We defined $e_{ij}=H_1(C_{ij})\cdot H_2(P_{ij})$ to represent the edge in $G(V, E)$ between gene i and gene j with

the prior association confidence level C_{ij} and the correlation p-value P_{ij}. $H_1(\bullet)$ and $H_2(\bullet)$ are Heaviside functions such that the significant edges in $G(V, E)$ were selected. In this study, we defined $H_1(C_{ij})=1$ if $C_{ij}\geq 0.7$; otherwise, $H_1(C_{ij})=0$. In addition, $H_2(P_{ij})=1$ if $P_{ij}\leq 0.05$; otherwise, $H_2(P_{ij})=0$. Note that $e_{ii}=0$ for any i.

Validation and comparison of the DryNetMC with other methods

To assess the effectiveness and accuracy of our developed dynamic modeling method, we synthesized a GRN that was composed with 5 nodes and structured with typical motifs, such as positive and negative feedback loops and crosstalk (**Fig S3A**).

The following ODEs were built to generate the original time course gene expression data,

$$\frac{dx_i}{dt}=\sum_{j=1}^{5}a_{ij}x_j+b_i\,,\quad (i=1,2,\cdots,5)\,. \tag{S8}$$

The interaction confidents (a_{ij}) were given in (**Fig S3B**) and the degradation rates (b_{ij}) were set to -0.1. The initial values of the above ODEs were set as random numbers subject to the uniform distribution on [0, 1]. By numerically solve the above ODEs we obtain a set of time series data, referred as the original simulated data (**Fig S3C**). Then we sampled data at 0, 6, 12, 24 and 48 hr to mimic the experimental measurements (as in our RNA-seq data). Based on the sampled data, we evaluated whether the dynamic modeling method in the DryNetMC can reconstruct the gene interaction edges in the true network.

Under the assumption that the gene expression trajectory is temporally continuous and piece-wisely monotone on each sampled time interval, the piecewise cubic Hermit interpolations were used to approximate the temporal gene expression profiles at more time points within 0 to 48 hr with interval of 1 hr (**Fig S3C**). The Hermit interpolations approximated well to the original data. The prior knowledge of the network was also assumed by randomly adding a set of additional edges to the true network.

We used the dynamic modeling method approach proposed in the DryNetMC (Figure 1 in the main text) to infer the network. The edge strength (i.e., the absolute value of the estimated interaction coefficient) was used as a score to predict the occurrence of an edge. True positive rate (TPR) and false positive rate (FPR) are defined by Equations (S9) and (S10) respectively:

$$TPR=\frac{TP}{TP+FN} \tag{S9}$$

$$FPR=\frac{FP}{FP+TN} \tag{S10}$$

where TP, FP, TN and FN are the numbers of true positives, false positives, true negatives and false negatives, respectively. TPR and FPR were used to plot the receiver operating characteristic (ROC) curves and the area under ROC curve (AUC) is calculated.

We also compared the DryNetMC with several state-of-the-art methods used for inferring GRNs, including PCC-based correlation network method (PCCNet) [9], tree-based ensemble learning methods (GENIE3 [10]), Bayesian network or dynamic Bayesian network methods (such as GRENITS (i.e., Gene Regulatory Network Inference Using Time Series) [11]), the ODE-LASSO method (OdeLasso) and the ODE modeling method incorporating prior network

information (OdeLassoP) [12, 13]. For the PCCNet method, the absolute value of the correlation coefficient of each pair of genes was used as a score to predict the occurrence of an edge. The correlation coefficient was set to 0 if the corresponding p-value is not less than 0.05. For the GENIE3, the default parameters and setting were used. For the GRENITS, the latest version of "GRENITS" package (1.34.0) was employed. We implemented "LinearNet" function in the package to infer the GRN, since the simulated gene expression data were generated from a linear ODEs model. The default parameters and setting were used. The inferred probability for each interaction was used as a score to predict the presence of an edge. For the OdeLasso, the similar procedures of ODE modeling and LASSO regression were performed as in the DryNetMC but without data interpolation and prior information incorporation. For the OdeLassoP, the prior network information was incorporated into OdeLasso method during network inference. The ROC curves and the AUC values were employed for comparing these methods. (**Fig S3D**).

Moreover, we compared the performance of the DryNetMC with the other methods based on more datasets. We synthetically generated 100 networks by randomly perturbing the above interaction confident matrix A=$(a_{ij})_{5X5}$ (listed in Fig S3B) for 100 times. For each time, we randomly selected one element in A and changed its value to be a random number sampled from a uniform distribution on [-0.1, 0.1]. The initial values of the systems (S8) used for simulating gene expression dynamics data were also randomly sampled from a uniform distribution on [0, 1]. By numerically solving the ODEs we obtain 100 sets of time-course gene expression data, referred as the original simulated data. Then we sampled data at 0, 6, 12, 24 and 48 hr to mimic the experimental measurements (as in the RNA-seq data). Based on the 100 sets of sampled data, we compared the performance of the related methods with respect to reconstructing the gene interaction edges in the true network. The AUC values for the DryNetMC and the other methods (i.e., PCCNet, GENIE3, GRENITS, OdeLasso and OdeLassoP) were calculated using all the 100 datasets for comparison (**Fig S4**). One-tailed Wilcoxon signed rank test p-values were used to assess the statistical significance of the difference between the AUC of the DryNetMC and that of the other methods.

Identifying DryNB for predicting drug-resistance of glioma patients

The differential network captures the robust topological difference between the sensitive network and resistant networks. Therefore, the differential network reflects the potential change in the gene regulations during the acquisition and development of drug resistance. The genes in the differential network should play important roles in promoting tumor growth even under the drug treatment condition. We hypothesized that the genes in the differential network are associated with the survival outcome of glioma patients who received targeted therapeutics. Therefore, we developed a differential regulatory network-based biomarker (DryNB) identification model to identify drug-resistance biomarker for glioma patients.

We collected the clinical information and RNA-seq gene expression data of glioma patients from TCGA database (https://cancergenome.nih.gov/). By matching both patient sample IDs and gene names from the clinical information and the gene expression data, a dataset (*n*=289) of glioma patients who received target therapy was prepared. The 3-year survival statuses (alive or dead) were defined as the outcome (sensitive or resistant) of targeted drug treatment.

Let y_i=0 or 1 denote the binary response outcome of the *i*-th sample of *n* individuals. Denote $p_i = \Pr(y_i = 1)$, $i = 1,\cdots,n$. $x_i = (x_{i1}, x_{i2}, \cdots, x_{iL})^T$ represents the expression levels of genes in the differential regulatory network for the *i*-th patient. The relationship of y_i with x_i was modeled through a logistic regression model as follows:

$$\text{logit}(p_i) = \log\left(\frac{\Pr(y_i = 1)}{1 - \Pr(y_i = 1)}\right) = \beta_0 + \sum_{j=1}^{L} \beta_j x_{ij}. \quad \text{(S11)}$$

where β_0 and β_j are the intercept and regression coefficient, respectively. Denote $\beta = (\beta_0, \beta_1, \cdots, \beta_L)$ and re-write $x_i = (1, x_{i1}, x_{i2}, \cdots, x_{iL})^T$, then the above logistic model can be formulated as $\text{logit}(p_i) = x_i^T \beta$.

We employed the following differential regulatory network-based weighted LASSO method to estimate the above parameters:

$$\beta^* = \arg\min\left(\sum_{i=1}^{n} \log\left(1 + \exp\left(x_i^T \beta\right) - y_i\left(x_i^T \beta\right)\right) + \lambda \|\omega \cdot \beta\|_{L^1}\right), \quad \text{(S12)}$$

where λ is the tuning parameter and ω is the weight vector measured by the ranking of the importance score (Equation (11) in the main text).

The genes corresponding to non-zero components in the estimated *β** at the optimal tuning parameter were selected as DryNB. The selected genes were $\hat{X}$ = (KIF2C, CCNA2, NDC80, KIF11, KIF23, ANLN, and CENPM), and the corresponding coefficients were $\hat{\beta}$ = (0.43219482, 0.61440130, 0.43219482, -1.09116226, 0.46333400, 0.06288534, -0.26516920). Therefore, the following drug-sensitivity score was formulated based on the expression levels of the DryNB genes: $S = \frac{e^{\hat{X}\cdot\hat{\beta}}}{1 + e^{\hat{X}\cdot\hat{\beta}}}$.

The performance of the DryNB for predicting the response of glioma patient to the targeted therapies was assessed with the area under the curve (AUC) of ROC. The TCGA samples of glioma patients were randomly divided into the training and test sub-datasets with several different sample ratios (50%, 60%, 70% and 80%) to the total sample number (*n*=289). The sample division at each ratio was repeated 100 times. The AUCs of ROC of the two methods were computed for both the training dataset and the test dataset.

Assessing the association of the DryNB genes with prognosis of glioma patients

The Cox PH model [14] was used to compute the risk scores for patients in the TCGA dataset based on the expression levels of the above 7 DryNB genes. The classification of patients into the high-risk and low-risk groups was based on the optimal cutoff value using the ROC method. The statistical significance of the difference between the Kaplan–Meier (K-M) curves for patients in two groups was assessed using the two-sided log-rank test.

Significance test of the prioritized genes

To test the statistical significance of the top 5 genes prioritized by the DryNetMC (i.e., KIF2C,

CCNA2, NDC80, KIF11 and KIF23), we used a bootstrapping method. The null hypothesis was that the DryNetMC-prioritized 5 genes were randomly selected. Define the statistics $S = D_S - D_R$, where D_S was the distance from the tested cells to the sensitive cells, and D_R was the distance from the tested cells to the resistant cells. The above distance was measured by the temporal pattern similarity of the selected genes. So $S_{obs} = d_S - d_R$, where d_S denotes the distance between the tested cells and the sensitive cells, and d_R the distance between the tested cells and the resistant cells, both evaluated by the DryNetMC-prioritized genes. We then randomly picked 5 DEGs (analyzed using DEseq2) and used them as biomarker to evaluate the distance from the tested cells to the sensitive cells ($\widetilde{D_S}$) or to the resistant cells ($\widetilde{D_R}$). So $S_{rand} = \widetilde{D_S} - \widetilde{D_R}$. This process was repeated for a large number of times (such as 1000 times). We then estimated the probability of $S_{rand} < S_{obs}$. If the probability $P(S_{rand} < S_{obs})<0.05$, then the null hypothesis was rejected, indicating non-randomness of the DryNetMC-prioritized 5 genes.

Supplementary references

1. Trapnell C, Pachter L, Salzberg SL. TopHat: discovering splice junctions with RNA-Seq. Bioinformatics. 2009;25(9):1105-11.
2. International Human Genome Sequencing C. Initial sequencing and analysis of the human genome. Nature. 2001;409:860.
3. Anders S, Pyl PT, Huber W. HTSeq—a Python framework to work with high-throughput sequencing data. Bioinformatics. 2015;31(2):166-9.
4. Anders S, Huber W. Differential expression analysis for sequence count data. Genome Biology. 2010;11(10):R106.
5. Dougherty RL, Edelman AS, Hyman JM. Nonnegativity-, monotonicity-, or convexity-preserving cubic and quintic Hermite interpolation. Mathematics of Computation. 1989;52(186):471-94.
6. Von MC, Huynen M, Jaeggi D, Schmidt S, Bork P, Snel B. STRING: a database of predicted functional associations between proteins. Nucleic Acids Research. 2003;31(1):258.
7. Szklarczyk D, Franceschini A, Kuhn M, Simonovic M, Roth A, Minguez P, et al. The STRING database in 2011: functional interaction networks of proteins, globally integrated and scored. Nucleic Acids Research. 2011;39(Database issue):561-8.
8. Szklarczyk D, Morris JH, Cook H, Kuhn M, Wyder S, Simonovic M, et al. The STRING database in 2017: quality-controlled protein–protein association networks, made broadly accessible. Nucleic Acids Research. 2017;45(Database issue):D362-D8.
9. Liu B-H. Differential Coexpression Network Analysis for Gene Expression Data. In: Huang T, editor. Computational Systems Biology: Methods and Protocols. New York, NY: Springer New York; 2018. p. 155-65.
10. Huynh-Thu VA, Irrthum A, Wehenkel L, Geurts P. Inferring Regulatory Networks from Expression Data Using Tree-Based Methods. PLOS ONE. 2010;5(9):e12776.
11. Morrissey E. GRENITS: Gene Regulatory Network Inference Using Time Series. R package version 1.34.0. 2018.
12. Christley S, Nie Q, Xie X. Incorporating Existing Network Information into Gene Network Inference. PLOS ONE. 2009;4(8):e6799.

13. Zheng Z, Christley S, Chiu WT, Blitz IL, Xie X, Cho KW, et al. Inference of the Xenopus tropicalis embryonic regulatory network and spatial gene expression patterns. BMC Systems Biology. 2014;8(1):3.
14. Cox DRJJotRSS. Regression models and life-tables. 1972;34(2):187-220.
