# Supplementary material for: Differential regulatory network-based quantification and prioritization of key genes underlying cancer drug resistance based on time-course RNA-seq data
Source: PLoS Comput Biol. 2019 Nov 4;15(11):e1007435. doi: 10.1371/journal.pcbi.1007435 (PMC6827891; doi:10.1371/journal.pcbi.1007435)
Supplement: S2 Text — The file includes the following sections: Cell culture and reagents, Western Blotting, qPCR and Morphological imaging. (PDF) [file pcbi.1007435.s014.pdf]

Supplementary Materials for

Differential regulatory network-based quantification and prioritization of key genes underlying cancer drug resistance based on time-course RNA-seq data

Jiajun Zhang[1#], Wenbo Zhu[2#], Qianliang Wang[1], Jiayu Gu[2], L. Frank Huang[3,4*], Xiaoqiang Sun[5,1*]

[1] School of Mathematics, Sun Yat-Sen University, Guangzhou 510080, China.

[2] Department of Pharmacology, Zhongshan School of Medicine, Sun Yat-Sen University, Guangzhou 510080, China.

[3] Brain Tumor Center, Division of Experimental Hematology and Cancer Biology, Cincinnati Children's Hospital Medical Center, Cincinnati, OH 45229, USA.

[4] Department of Pediatrics, University of Cincinnati College of Medicine, Cincinnati, OH 45229, USA.

[5] Department of Medical Informatics, Zhongshan School of Medicine, Sun Yat-Sen University, Guangzhou 510080, China; Key Laboratory of Tropical Disease Control (Sun Yat-Sen University), Chinese Ministry of Education, Guangzhou 510080, Guangdong, China.

* To whom correspondence should be addressed. Tel: 86-020-87330128; Fax: 86-020-87330420; Email: sunxq6@mail.sysu.edu.cn or xiaoqiangsun88@gmail.com (XS; Lead contact); Frank.Huang@cchmc.org or hleicug@gmail.com (LH)

Text S2 Supplementary experimental methods

Cell culture and reagents

The cell lines including DBTRG-05MG, U87MG, and LN-18 were purchased from the American Type Culture Collection. DBTRG-05MG, U87MG, and LN-18 cells were respectively maintained at 37℃ under 5% CO_2 in RPMI1640, MEM or DMEM supplemented with 10% fetal bovine serum (Gibco) and penicillin/streptomycin. dbcAMP (100 mM, dissolved in double distilled water [ddH2O], Sigma-Aldrich) was used as a reagent in this study.

Western Blotting

Cells were lysed using M-PER mammalian protein extraction reagent (Thermo Scientific), followed by SDS-PAGE. After being electroblotted onto a polyvinylidene fluoride membrane (Roche), GFAP protein was detected with corresponding antibodies: human GFAP (GB11096, Wuhan Servicebio Technology CO.,LTD) and human α-Tubulin (Arigo).

qPCR

Total RNA was extracted using TRIzol (Life Technologies) reagent and reverse-transcribed to cDNA using oligo(dT). Specific gene expression was quantified with SuperReal PreMix SYBR Green (Geneseed Biotech Co.,Ltd.) using an Applied Biosystems 7500 fast real-time PCR system (Life Technologies, Applied Biosystems). The following amplification primers (Sangon Biotech (Shanghai) Co.,Ltd.) were used (50 to 30):

CCNA2 (Forward, TACTTTCTGCATCAGCAGCCT; Reverse, AGCTTTGTCCCGTGACTGTG),

KIF11 (Forward, CAACAGGTACGACACCACAGA; Reverse, AGCATCATTAACAGCTCAGGCT),

KIF23 (Forward, TTCTCCATCACCTGTGCCTC; Reverse, GCTGCTGCAAGAGTTAGAGC),

NDC80 (Forward, GCAGAGTATCACAAATTGGCTAGA; Reverse, TTTGACAAGGCAGTTGGCAC), KIF2C (Forward, GGACTTGCATGATTGCCACG; Reverse TCAGCTCCTTGACCCTGTCT).

Morphological imaging

Cells were treated with 1 mM dbcAMP for indicated times, and then were imaged under a microscope (Nikon ECLIPSE Ti-U).
